# Forward Genetics in Apicomplexa Biology: The Host Side of the Story

**DOI:** 10.3389/fcimb.2022.878475

**Published:** 2022-05-12

**Authors:** Juan C. Sánchez-Arcila, Kirk D. C. Jensen

**Affiliations:** ^1^ Department of Molecular and Cell Biology, University of California Merced, Merced, CA, United States; ^2^ Health Science Research Institute, University of California, Merced, Merced, CA, United States

**Keywords:** RNAi, CRISPR-Cas9, ENU, forward genetic screens, apicomplexa, host immunity, QTL, classical genetics

## Abstract

Forward genetic approaches have been widely used in parasitology and have proven their power to reveal the complexities of host-parasite interactions in an unbiased fashion. Many aspects of the parasite’s biology, including the identification of virulence factors, replication determinants, antibiotic resistance genes, and other factors required for parasitic life, have been discovered using such strategies. Forward genetic approaches have also been employed to understand host resistance mechanisms to parasitic infection. Here, we will introduce and review all forward genetic approaches that have been used to identify host factors involved with Apicomplexa infections, which include classical genetic screens and QTL mapping, GWAS, ENU mutagenesis, overexpression, RNAi and CRISPR-Cas9 library screens. Collectively, these screens have improved our understanding of host resistance mechanisms, immune regulation, vaccine and drug designs for Apicomplexa parasites. We will also discuss how recent advances in molecular genetics give present opportunities to further explore host-parasite relationships.

## Introduction

### Apicomplexa and the Utility of Genetic Screens

The phylum Apicomplexa is an exclusive group of unicellular protozoa able to infect most species of warm-blooded animals, such as humans, birds, and rodents, as well as terrestrial and marine invertebrates. Between 1-10 million species of Apicomplexa are estimated to exist (Adl et al., 2007), including the medically important *Babesia* spp., *Cyclospora* spp., *Cryptosporidium* spp., *Cystoisospora* spp., *Plasmodium* spp., and *Toxoplasma gondii* (Votýpka et al., 2017). Apicomplexan species are defined by their unique invasion machinery present in the apical portion of their cell (Guizetti and Frischknecht, 2021) and apart from Apiroplasmida, most apicomplexans have a lytic cycle that includes a parasitophorous vacuole required for intracellular life (Coppens and Romano, 2020). Apicomplexan parasites are highly diverse and can invade several types of cells such as red blood cells, leukocytes, neurons and enterocytes. Spread between hosts is likewise varied, including transmission by arthropods, or by oral consumption of infective cysts or oocysts. Such diversity in host range and cell types infected provides a unique opportunity to explore eukaryotic immunity in a variety of settings.

Genetic screens are a powerful set of techniques widely used to identify genes responsible for an observed phenotype. Genetic screens applied to host-parasite interactions have yielded important findings, including how the genetic background of an organism influences their fitness to infection and the microbial genes are required for parasitic life. At its core, forward genetic screens associate function to genes in an unbiased way. They do so by taking advantage of a genetically diverse population of hosts or cells to make genotype to phenotype correlations, ideally leading to gene discovery and function. Forward genetics screens have been successfully leveraged to identify genes responsible for encoding the parasite’s molecular machinery required for growth and virulence. A complete set of reviews and book chapters have been written to describe the history and contributions of forward genetics to study the “parasite side” of the story (Balu, 2012; Behnke et al., 2016; Egan, 2018; Huang et al., 2018; Damena et al., 2019; Behnke et al., 2020).

In the present discussion, however, we will review the impact that forward genetic approaches have had on the ‘host side’ of the story. We will discuss how forward genetic screens have revealed novel host resistance mechanisms and pointed to new strategies to control infections by apicomplexan parasites. We do not imply that forward genetics is the best way to study host responses. Indeed, many approaches are better suited for addressing directed questions, cell biological and biochemical studies notwithstanding. Rather the allure of forward genetics screens is that they may unexpectedly reveal novel genetic information and putative host mechanisms not yet considered in host immunity to Apicomplexa parasites.

### An Introduction to Genetic Screens

Genetic screens are separated into two general categories of ‘reverse’ and ‘forward’ genetics. The basic principle in reverse genetics (‘gene-driven’ or ‘gene to phenotype’ approaches) is to evaluate the functional effect of a specific gene through modification or deletion (Orkin, 1986). In reverse genetics, the gene is chosen based upon *a priori* understanding or prediction of its function in the host-parasite interaction. Once the gene sequence is well characterized, the gene can be targeted to change its expression or deleted. In this way, it is straightforward to verify the association between the change induced in the gene and an observable phenotype precisely.

Forward genetic screens (FGS), in contrast, start with a phenotype for which the causal genes underlying that function are undetermined. Within a forward genetic approach exist a variety of tools and screening techniques that can pinpoint the polymorphic or mutant gene responsible for the phenotype. The first forward genetics screens implemented what is referred to as the ‘classical genetics’ approach. Classical genetics is based on crossing individuals that differ in their phenotype, say resistance to infection, and verifies the heritability of this trait in their progeny. This approach relies on a genetic linkage map and can identify genomic loci harboring a gene of interest. Today, fully sequenced genomes of the parents or progeny greatly assist the identification of the causal gene responsible for the trait in question. A critical feature of FGS is its unbiased nature. Because FGS rely on natural or induced genetic differences between hosts of varying phenotypes, it does not require a specific working hypothesis about the studied trait to explain how an underlying gene(s) may function to generate the phenotype. This is an advantage because apicomplexans interact with a multitude of animals and cell types, making it challenging to know which immune mechanisms are at play in each of these contexts.

### Types of Forward Genetic Screens

**Figure 1:**
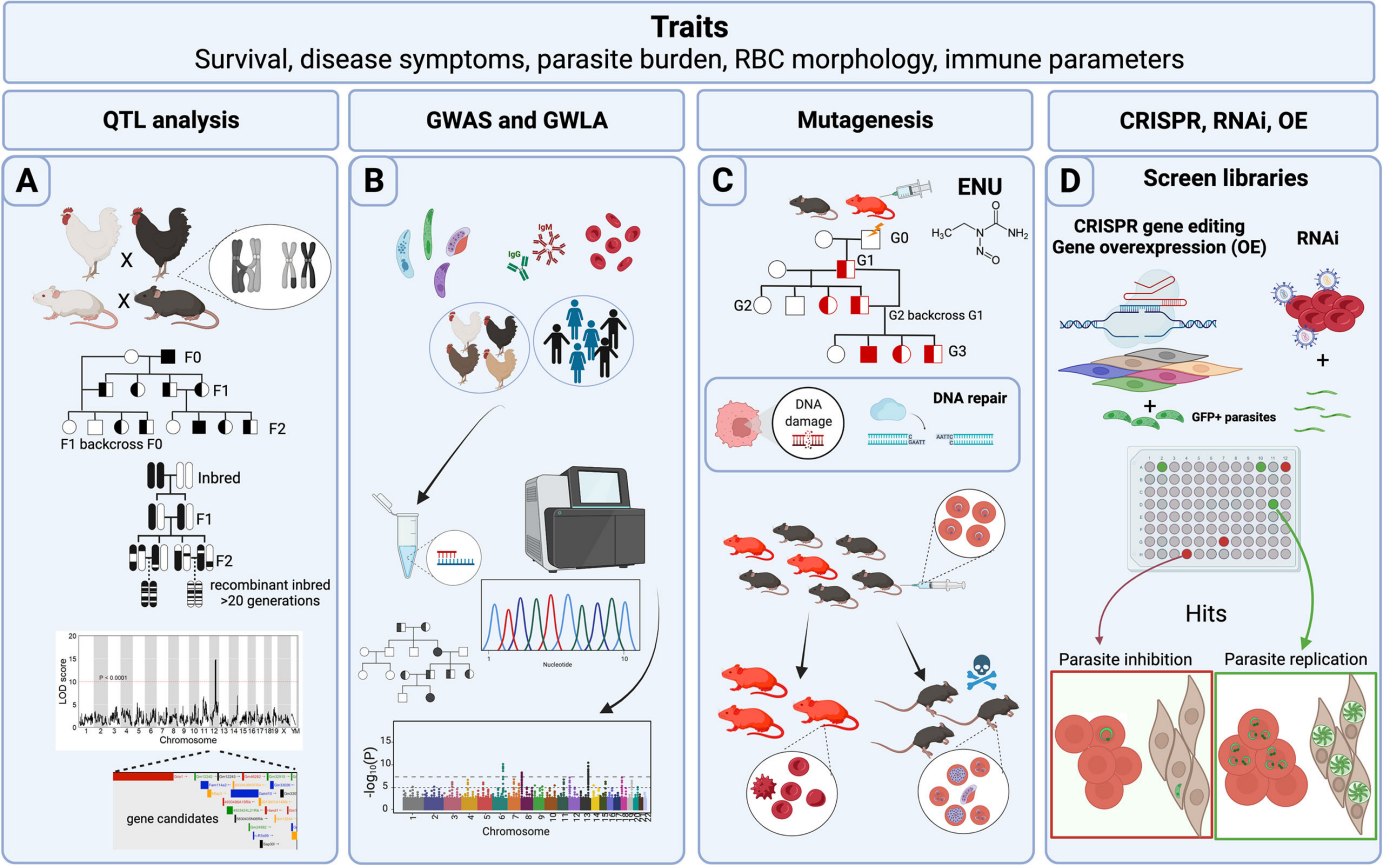
Host forward genetic screens in Apicomplexa. Common traits measured in forward genetic screens (FGS) are survival to Apicomplexa infection, symptoms related to the disease such as fever or tissue pathology, prevalence of the disease within a population, parasite load reduction, variables such as red blood cell (RBC) morphology and physiology, and immune parameters such as cytokines and parasite-specific antibody titers. **(A)** Classical genetic approaches use crosses between different genetic backgrounds that differ in susceptibility to infection. Rodents, pigs, and chickens have been analyzed for quantitative trait loci (QTLs) following Apicomplexa infection. In addition, more complex crosses of mice including backcrosses of murine recombinant inbred lines (RILs), or more advanced populations of RILs have been used to generate QTLs with narrow regions of association. The final objective is to map genomic regions and identify variants associated with the trait. Data is frequently represented as a QTL map showing peaks over the genetic regions with highest trait association. Candidate genes are usually indicated within the QTL. **(B)** Genome-wide association studies (GWAS) and genome-wide linkage analysis (GWLA) seek genotype-phenotype correlations for a variety of traits associated with disease caused by Apicomplexa; humans and chickens have been subjected to these approaches. These screens rely on a high quantity of genetic markers, or SNPs that are determined by differential oligo binding or whole genome sequencing of individuals enrolled in the study. Associations are visually displayed using Manhattan Plots, showing strength of the probability (-Log10(P)) and the chromosomal localization of the SNPs. **(C)** ENU mutagenesis screens are designed to generate 1 mutation in every 700 loci and relies upon small failures of the host’s DNA repair machinery. Mutagenesis screens start with a male treated with ENU and crossed with healthy females. Various breeding schemes can be used to segregate the gene causative of the induced trait (*e.g.* protection against *Plasmodium* infection). **(D)** CRISPR, RNAi, and gene overexpression (OE) library screens are adapted for high-throughput *in vitro* conditions, and can screen hundreds or thousands of genes during the process. CRISPR and RNAi inhibit while OE enhances gene function through overexpression. GFP+ parasites are often used to measure parasite proliferation to help identify host genes capable of inhibiting or promoting parasite replication and growth. Credits: Figure created in Biorender.

Genomic variability is a necessary condition to perform FGS. Variation that occurs naturally in a population can be enough fodder for discovery of a gene’s function. However, different approaches have been used to increase the genetic diversity of a population, expediting discovery of gene function. We find it useful to briefly discuss these approaches prior to delving into forward genetic screens for the study of host-Apicomplexa interactions (**Figure 1**).

#### Natural Variation and Genetic Linkage

Classical genetics capitalizes on the genetic basis of inheritance and meiosis. Meiosis, which creates “chimeric chromosomes” between maternal and parental chromosomes (**Figure 1A**), randomly separates allelic variants of two separate genes at the point of crossover. Since some genes do not have an independent pattern of chromosomal segregation because they exist on the same chromosome, the importance of gene positional mapping is paramount. Importantly, the degree of “linkage”, or lack of meiotic crossover events between any two genes on a single chromosome, increases as a function of the physical proximity between genes on a chromosome. By crossing individuals with two different phenotypes, one can trace whether the genes responsible for the two phenotypes localize to the same chromosomal region, making it possible to create ‘linkage maps’ (Sturtevant, 1913). With the availability of more precise genetic maps, phenotypic traits can be linked to positions within the genome, thereby generating quantitative trait loci (QTL). Several types of breeding programs for specific trait evaluations (F1, F2, intercrossing, backcrossing, and others) (**Figure 1A**) have been used to promote the discovery of candidate genes and their genetic location. Still, the evaluation is not always straightforward. Even with crucial factors such as genetic variability and precise genetic maps, the resolution at a genetic locus for a given phenotype may be confounded by several factors. For example, as the number of genes controlling a phenotype increases, the power to detect causal genes decreases. Moreover, inherent variation within experimental systems and the limited number of progeny bearing meiotic cross-over events in a region of interest, which depends on the recombination frequency at a given locus, can impede genetic resolution and genotype-phenotype correlations. For these reasons, large and diverse populations are required to assist in locating causal genes within a QTL.

To overcome QTL resolution problems in mammalian classical genetics, recombinant inbred lines (RIL), such as the BXD product of crossing between C57BL/6J and DBA/2J mice, and other advanced intercross lines, such as the Collaborative Cross (CC) and the Diversity Outbred (DO) panels (Churchill et al., 2012; Srivastava et al., 2017), have been generated to help researchers improve resolution of genetic loci with natural variants. One of the main advantages of these breeding programs is that compared to F2 populations, the resultant progeny have an increased number of chromosomal crossover events due to the multiple rounds of breeding required to generate these panels (**Figure 1A**), which greatly aids in refining QTL boundaries. In the case of RIL and CC panels, alleles are bred to homozygosity, fixing crossovers and aiding phenotypic penetrance of recessive alleles. The lack of genotyping needed to test individuals within an RI panel saves both time and economic resources during the mapping process and reproducibility can be confirmed between different labs analyzing the same RIL. The Collaborative Cross is a multi-parental recombinant inbred panel of mice created from eight founder parental strains. By including in its founders’ contributions from three different subspecies of mice (*musculus, castaneus, domesticus*), the CC panel captures >90% of the estimated genetic diversity of *Mus musculus*, expanding the potential to find novel allelic variants. The CC may also best support integrative, systems-level biology or ‘systems genetics’ in mice to uncover biological networks revealed by novel alleles and phenotypes expressed in the panel (Threadgill and Churchill, 2012; Srivastava et al., 2017). Finally, the Diversity Outbred is the newest advanced intercross panel and was derived from individual CC founders. It maintains both high genetic diversity, but, unlike RILs, seeks allelic heterozygosity, keeping a genetic balance of the founder genomes but avoiding allelic loss. No two individuals within the DO panel are identical, providing an opportunity to capture even more genetic admixture than the CC panel.

#### GWAS and GWLA

Due to the increased availability of genomic sequences during the last two decades, genome-wide association studies (GWAS) constituted the most common forward genetics technique used to study human genetic associations with diseases caused by Apicomplexa species. The advantage to GWAS is that controlled genetic crosses or induced mutational screens are not required to make genotype-phenotype correlations in an analyzed population of humans. In GWAS, small nucleotide polymorphisms (SNPs) are assessed either by large scale oligo hybridization assays or by whole genome sequencing and correlated with a particular trait by scanning genome-wide markers for association in individuals expressing the trait of interest (Visscher et al., 2012). Like GWAS, genome-wide linkage analysis (GWLA) makes similar SNP trait correlations but takes advantage of human pedigrees to understand the inheritance of genetic variants in a particular family or community (Ott et al., 2015; Modena et al., 2019). GWLA has proven effective in refining genomic regions associated with Mendelian inheritance traits but often lacks the power to study complex traits, such as complex genetic disorders (**Figure 1B**).

The study of natural genetic diversity in classical genetics relies upon polymorphisms that may not alter the gene function drastically enough to reveal its identity, a characteristic that can delay the discovery of genes responsible for complex traits. Many FGS approaches have been taken to increase the genetic diversity of a population artificially. Most of the induced changes cause drastic changes to a gene’s function, thereby increasing the likelihood of finding the causal gene in a screen. Below are approaches taken to induce artificial variation in organisms used for genetic screens.

#### Radiation

The first description of radiation used as a potent mutagenic was conducted by Herman Joseph Muller in *Drosophila* sp. (Muller, 1928). His research regarding the cause of mutations by X-rays was awarded the Nobel Prize in Physiology and Medicine in 1946. Since then, ionizing radiation such as α, β, and γ-rays have been used as a mechanism to induce mutations in several organisms, from plants to mice. The mutagenic effect of ionizing radiation lies in its ability to induce double strand DNA breaks. Although organisms have very efficient DNA-repairing systems, these mechanisms are not always perfect, creating changes to the original DNA sequence during repair. Everything from small scale deletions, insertions and point mutations, as well as larger DNA chromosomal inversions and segment removal can ensue following repair of the genetic lesion. Such variants or ‘mutants’, can then be used for classical genetic screens (Rinchik et al., 1991).

#### Chemical Mutagenesis [N-Nitrosourea (ENU)]

The aim of ENU mutagenesis is like that of radiation, however, the types of genetic changes are more uniform as ENU introduces random point mutations throughout the genome. This strategy is known for generating null alleles, partial loss-of-function, or generating alleles with novel functions (Acevedo-Arozena et al., 2008), which can then be studied to verify their effect for a specific phenotype. The primary mechanism of action of ENU is by transferring its ethyl group to oxygen or nitrogen radicals in DNA, which when repaired generates a SNP (**Figure 1C**). When ENU is administered to male animals, the highest number mutations occur in premeiotic spermatogonial stem cells, which can then be passed to their progeny (Justice et al., 1999). Typically, in an ENU mutagenesis screen, ENU treated males are bred with wild-type females, and then the F1 progeny is further bred and subjected to classical genetic screening. Since its discovery in the late 1970s (Russell et al., 1979), ENU mutagenesis has recently gained ‘fresh air’ because of new genome sequencing technologies that can quickly lead to positional SNP identification by comparing the mutagenized to the parental genome.

#### Overexpression

Gene overexpression is used to modify cells to express genes and their protein products in quantities above normal levels (Prelich, 2012) (**Figure 1D**). Typically, these screens are not genome-wide and usually rely on the use of a pool of genes collected from cDNA synthesis and eventually expressed in a cell line. The first clues that gene dosage could impact the normal functioning of an organism came from observations of certain human genetic syndromes (aneuploidy) and other plant or animal mutants (aneuploidy, polyploidy) that were caused by the presence of an abnormal number of chromosomes. The first overexpression screens utilized plasmid libraries to transform yeast to study mechanisms of drug resistance (Rine et al., 1983), and reviewed by Rose & Broach in (Rose and Broach, 1990), and genes that contributed to chromosomal segregation defects (Meeks-Wagner and Hartwell, 1986). A complete review of the historical development of overexpression screens was written by Gregory Prelich and can be found in (Prelich, 2012).

#### RNAi

The discovery of small non-coding RNA sequences of ~20-30 nucleotides with the power to regulate gene expression launched a new chapter in molecular biology. Andrew Fire and Craig Mello’s groundbreaking discovery opened the possibility of using sequence-specific gene knockdown strategies to block protein expression in targeted cells as a therapeutic approach in human disease, including inhibiting viral infection (Fire et al., 1998), reviewed by Wilson and Doudna in (Wilson and Doudna, 2013). The programable nature of this RNA suppression pathway, present in many eukaryotic organisms, permitted the use of interference RNA (RNAi) across the genome, facilitating fast, high-throughput cell-based screening studies, with incredible potential to detect therapeutic targets or infectious disease resistance factors (Fernandez-Cortes et al., 2017; Wang et al., 2020) (**Figure 1D**). Though RNAi was initially implemented as a reverse genetics technique, it was quickly encompassed within the forward genetics toolkit, effectively allowing reverse genetics genome-wide.

#### CRISPR/Cas9

One of the mechanisms that bacteria employ to defend themselves against foreign plasmids and virus invasion is based on the transcription of clustered regularly interspaced short palindromic repeats (CRISPR) that associate with a CRISPR-associated (Cas) endonuclease to mediate RNA-guided DNA cuts at sequence-specific sites in foreign DNA (Jiang and Doudna, 2017) (**Figure 1D**). The high specificity used by bacterial CRISPR-Cas systems to target specific DNA sequences and their remarkable plasticity were successfully manipulated by the pioneering work of Doudna and Charpentier to develop an efficient, versatile, and programmable system to edit and modify any genomic sequence (Jinek et al., 2012). Like overexpression and RNAi, CRISPR-Cas belongs within the reverse genetics toolkit, but has been successfully used in high-throughput FGS to reveal key factors for parasite replication and dissemination (Sidik et al., 2016; Sangaré et al., 2019).

Forward genetics has a rich history in biology and has contributed to significant discoveries in genetics, such as the mapping of the gene *HD*, responsible for Huntington’s disease (MacDonald et al., 1993), or the gene *Clock*, involved in circadian rhythm alteration (Vitaterna et al., 1994). Forward genetics has similarly made notable contributions to immunology research. For example, the identification of *Tlr4* as the gene involved in recognition of bacterial LPS in mammals (Poltorak et al., 1998), *Tlr9* involved in recognition of pathogen-derived molecules (Tabeta et al., 2004), and the discovery of *Irgb10* as a GTPase capable of mediating the inhibitory effect of IFNγ (Bernstein-Hanley et al., 2006), have each been described using forward genetics approaches. *Anopheles* spp. are vectors for malaria and necessary for *Plasmodium* development because the parasites start the sexual recombination in their gut. This review did not include studies of forward genetics applied to *Anopheles* or other invertebrates that are necessary for the life cycles of many hematozoa apicomplexans, and we consider they deserve a separate review due to the great quantity and richness of articles in this field. We strongly encourage entomologist colleagues to compile this valuable information in the future.

### Host Forward Genetics in Apicomplexa

Regarding the host side of the story, the first FGS involved classical genetics to reveal host loci and genes associated with susceptibility or resistance to Apicomplexa infections. Genetic linkage and QTL mapping have been widely used in *Plasmodium* sp., *T. gondii*, *Eimeria* sp., and *Sarcocystis* sp. infection studies. It is not uncommon to find reports of QTL regions without mechanistic proof of a gene candidates’ effect. In our review, we will discuss the important candidates within regions when appropriate, in full recognition of plausible versus proven mechanisms. In the last two decades, FGS has expanded to include genome-wide association studies, mutagenesis, and gene expression screens for host responses to Apicomplexa. We have chosen to separate our review based on FGS approaches and then within the approach, how it was used for a given parasite.

### Experimental Crosses, Linkage Analysis and QTLs

#### Toxoplasma gondii

*Toxoplasma gondii* is a parasite able to infect almost all nucleated cells in warm-blooded vertebrates. This plasticity explains why *T. gondii* has a worldwide distribution, reaching up to 60% of seropositivity in some places of the world. *T. gondii* can severely affect children during gestation, generating chorioretinitis, micro or macrocephaly, or inducing spontaneous abortion of the fetus. *T. gondii* can also affect immunocompromised individuals, including individuals receiving organ transplants, oncologic treatments, or those with AIDS. In addition to human infections, *T. gondii* uses cats as a definitive host to shed highly infectious oocysts that promote transmission and infection to animals of economic interest, such as pigs, sheep, goats, and birds (Hill et al., 2005). Since *T. gondii* can infect a broad host range, certain species and individuals within a population can be susceptible to infections, hence classical genetic screens have attempted to resolve the genetic basis for primary, chronic, and secondary infection susceptibility and resistance. While most vertebrates can be infected by *T. gondii*, murine models have been preferred. *Toxoplasma* immunological research has largely been performed in susceptible C57BL/6 mice, but other strains such as B10.D2, A/J and BALB/c have been used to study resistance. In particular, the AxB,BxA genetic cross between A/J and C57BL/6 have been useful to decipher genomic regions and genes associated with protection against severe infection.

The first classical genetic experiments in *T. gondii* were used to define the genetics of susceptibility of inbred mouse lines to primary infection (Williams et al., 1978). In the initial work, an analysis of F2 progeny from a cross between susceptible C57BL/6J and resistant B10.D2 mice suggested an association of susceptibility to *T. gondii* with *H-2* and *H-13* loci. Later Confirmation came later by McLeod et al. and colleagues (McLeod et al., 1989) using the AxB/BxA RIL panel derived from susceptible C57BL6/J and resistant A/J mice. Whereas no susceptibility QTLs to primary oral infection with the type II ME49 strain were initially identified, there was an association between resistance to chronic infection and the H-2a haplotype encoded within A/J mice. This locus was later dissected with precise H-2a congenic mice which revealed the MHC class I L^d^ molecule was responsible for promoting host resistance to cyst numbers during chronic infection (Brown et al., 1995). These observations were helpful for later discoveries that demonstrated the L^d^ MHC I molecule, which can bind longer peptides in its peptide binding groove, was uniquely responsible for presentation of an immuno-protective 10-mer C-terminal epitope from the dense granule GRA6 antigen to CD8 T cells (Blanchard et al., 2008). That certain alleles of the MHC I are better suited to facilitate cyst reduction is consistent with the known role that CD8 T cells play in controlling chronic infection (Suzuki, 2020).

In 2003, (B10.Q/J x BALB/c) x B10.Q/J F1 backcross mice were used to map an autosomal recessive gene observed in B10.Q mice propagated in Jackson but not Taconic laboratories. This allele rendered Jackson B10.Q mice unresponsive to IL-12 stimulation and unable to mount early IFNγ responses to *T. gondii* primary infection (Yap et al., 2001). Genetic mapping found the gene *Tyk2*, a kinase that signals downstream of the IL-12 and type I IFN receptors was unexpressed in B10.Q/J mice (Shaw et al., 2003). The authors demonstrated that, in the absence of this critical signaling molecule, the early IL-12 response required for Th1 immunity to *T. gondii* was dependent on Tyk2 and therefore led to early susceptibility to infection.

Like mice, strains of laboratory rats show differential susceptibility to infection during primary infection. Using genetic crosses between LEWxBN and LWxF344 rat strains, a locus called *Toxo1*, was associated with resistance against the type II Pru *T. gondii* strain (Cavaillès et al., 2006). Later, the same group and Cirelli and colleagues would describe *Nlrp1a*, located in the *Toxo1* locus, as the gene responsible for mediating rat resistance to infection (Cavailles et al., 2014; Cirelli et al., 2014). The NLRP1 inflammasome is best known for being cleaved by anthrax lethal toxin (LT) leading to pyroptotic death of host cells (Boyden and Dietrich, 2006; Chavarría-Smith and Vance, 2015). Macrophages from *T. gondii-*resistant Lewis rats underwent pyroptotic death following parasite infection, creating an inhospitable environment for parasite replication, while the converse was true for macrophages from susceptible rats (Shaw et al., 2003; Cirelli et al., 2014). This phenotype is NLRP1-dependent and could be transferred between rat macrophages with specific *Nlrp1a* alleles (Cirelli et al., 2014). Interestingly, whereas Lewis rats are sensitive to *T. gondii*-induced pryoptosis, they are resistant to LT-mediated pyroptosis, and conversely, *T. gondii*-resistant rats are sensitive to LT. *Nlrp1a* rat alleles differ by 20 NS-SNPs, and it is not clear how these polymorphisms produce these disparate outcomes but does suggest rodent *Nlrp1* is undergoing selection pressure by pathogens. Corroborating studies demonstrated mouse and human NLRP1 as mediators of *T. gondii* detection, immune regulation, and cell-autonomous immunity. For example, in mice the NLRP1 inflammasome detects *T. gondii* infection (Ewald et al., 2014; Gorfu et al., 2014) and contributes to *in vivo* IL-18 production, a required cytokine for optimal IFNγ secretion from NK cells and other cell types needed for immunity to *T. gondii* (Cai et al., 2000). In humans, congenital toxoplasmosis has been linked to *NLRP1* polymorphisms and *NLRP1* knockdown experiments found an association with premature host cell death following *T. gondii* infection (Witola et al., 2011). It is not clear why knockdown of NLRP1 would lead to enhanced pyroptosis in this context, but may suggest cross talk between human NLPR1 and other inflammasomes or cell death pathways. Finally, the finding that NLPR1 was a key sensor of *T. gondii* led to the later discovery that the *T. gondii* dense granules GRA35, GRA42 and GRA43 act as regulators of rat NLRP1 activation (Wang et al., 2019), showing the utility of genetic mapping experiments to open new fields of investigation for both host and parasite.

Although *T. gondii* can infect any cell, its preferred cell type *in vivo* are macrophages (Jensen et al., 2011), representing a key battle ground for host-*T. gondii* interactions (Park and Hunter, 2020). Hence, gene expression QTL (eQTL) analysis, which treats gene expression as a quantitative trait and is based on transcriptional data, was used to understand the genetic bases for differential macrophage responses to inflammatory stimuli and *T. gondii* infection (Hassan et al., 2015). Transcriptomes were evaluated for bone marrow-derived macrophages from 26 mice of the AxB/BxA RIL panel infected with *T. gondii* or stimulated with different cytokines or TLR agonists. The genetic mapping produced 2194 significant eQTLs, half of which mapped in *cis* to where the gene was encoded. Statistical support was found for the majority of *cis*-mapping eQTLs bearing polymorphisms within two kbs of their transcriptional start site (TSS), suggesting that a large fraction of differentially responsive genes may be explained by relative differences in transcription factor binding to polymorphic binding sites, as previously implicated (Keane et al., 2011). Examples of *cis*-mapping genes and functions included arginase expression and urea production in response to IL-4 stimulation, parasite growth and GBP (guanylate binding protein) expression in macrophages stimulated with IFNγ and TNFα. GBPs are IFNγ-induced GTPases that are important in vacuolar and parasite plasma membrane destruction (Saeij and Frickel, 2017). *Gbp1* and *Gbp2* gene expression and parasite restriction were more pronounced in resistant A/J macrophages and these phenotypes mapped to the GBP locus on chromosome 3. Additionally, the remaining eQTLs mapped in *trans* and most could be grouped into 25 *trans*-eQTL ‘hotspots’, whereby multiple eQTLs mapped collectively to a single locus. Further informatics analyses of genes defining the *trans*-eQTL hotspots on chromosomes 12 and 15 led to the identification of the transcription factor *Ddx1* as a repressor of nitric oxide production and the TLR signaling adaptor *Irak4*, as regulators of these *trans*-eQTLs, respectively. *Ddx1* and *Irak4* are polymorphic genes encoded within the *trans*-hotspots and mapped in *cis* after stimulating macrophages with IFNγ and TNFα. Modulating their expression through siRNA regulated many of the genes belonging to the *trans*-eQTL hotspots, potentially explaining A/J versus C57BL/6 macrophage response differences to stimulation by these cytokines. In conclusion, the enhanced arginase response which favors parasite growth (Jensen et al., 2011), combined with enhanced *Ddx1*-mediated suppression of nitric oxide, a static inhibitor of *T. gondii* (Adams et al., 1990; Hayashi et al., 1996; Scharton-Kersten et al., 1997), and lower GBP expression by macrophages from the susceptible background may contribute to survival differences between C57BL/6 and A/J mouse strains following primary infection.

RILs can also define genetic determinants of immunological memory responses. Recently, we reported in Souza et al. the use of the AxB/BxA RIL panel to study resistance determinants after secondary infections with highly virulent strains of *T. gondii* (Souza et al., 2021). Previously, we noticed that after vaccination or natural infection, C57BL/6 mice were unable to control secondary infections with most strains that express the virulence factors ROP5 and ROP18, which antagonize murine IRGs (immunity related GTPases) aimed at vacuolar destruction (Jensen et al., 2015). In contrast, A/J mice were protected from such challenges demonstrating a superior immunological memory response. Genetic mapping revealed four additive QTLs on chromosomes 7, 10, 11, and 17 that conferred survival to secondary infection. One of the most polymorphic gene candidates in the chromosome 7 QTL was *Nfkbid*, a gene that encodes IκBNS, a member of the atypical NF-κB inhibitors (Schuster et al., 2013). To test the hypothesis that *Nfkbid* was required for secondary infection immunity to *T. gondii*, we used *Nfkbid*-null ‘*bumble*’ mice, which were derived from an earlier ENU mutagenesis screen for genes controlling humoral responses to model antigens (Arnold et al., 2012). *Bumble* mice are known to lack fetal-derived innate-like B-1 cells and natural IgM production, which are important in the defense against flu and bacterial infections (Baumgarth, 2016). We found that *Nfkbid*-null mice survived primary infection with a low virulent type III strain but failed to survive a secondary infection with virulent type I strains, suggesting a major defect in the immunological memory response to *T. gondii*. It was discovered that B-2 cells in *Nfkbid*-null mice had major defects in maturation and could not produce parasite-specific IgM and generate robust anti-*T. gondii* IgG responses. Though B-1 cells contributed marginally to the high-affinity repertoire of antibodies to the parasite, they were absolutely needed for 100% vaccine efficacy to virulent challenge, improving immunity in bone marrow chimeric mice that lacked B-1 cells from 50% to 100% when B-1 cells were present. Interestingly, the polymorphism likely conferred relative differences in *Nfkbid* gene expression between mouse strains, which impacted plasma cell development and IgG1 responses against the parasite. Another key finding from this study was that in the resistant genetic background of A/J mice, both B-1 and B-2 cells underwent massive isotype class switching compared to the blunted responses observed in C57BL/6 mice. Hence, it is possible that the contribution of humoral immunity to apicomplexan parasites is underestimated in the commonly studied C57BL/6 mice.

#### *Plasmodium *sp.

*Plasmodium* spp. are the etiologic agents of malaria in humans, primates, rodents, birds, and reptiles. *Plasmodium* parasites can infect hepatocytes and red blood cells to differentiate and use a mosquito vector to transmit and complete its sexual cycle. In 2019, 409,000 malaria deaths were reported, caused mainly by *P. falciparum* and *P. vivax*, the most life-threatening malaria species in humans (WHO, 2019). Genetic data in human populations have shown associations between malaria resistance and polymorphisms in genes that cause sickle cell anemia, α-thalassemia, and G6PD deficiency, all of them impacting erythrocyte function. As red blood cells are the target of *Plasmodium* merozoites, many forward genetic screens have focused on detecting cell receptors used by the parasite to invade these cells or to discover genes that can change structural properties of the erythrocytes, inhibiting parasite replication.

Five species of *Plasmodium*: *P. ovale*, *P. malariae*, *P. knowlesi*, *P. vivax*, and *P. falciparum*, are recognized for causing human malaria. Like humans, rodents can be infected by a diverse group of *Plasmodium* parasites. Some species of *Plasmodium* such as *P. berghei*, *P. yoelii*, and *P. chabaudi*, have been used to investigate distinct aspects of malaria infections in rodents that mimic certain aspects of human disease, for example, cerebral malaria, placental malaria, liver injury, and blood stage infection. The importance of murine models is widely appreciated, and several reviews have addressed how mouse models have aided our understanding of human malaria infections (Craig et al., 2012; Zuzarte-Luis et al., 2014). Forward genetic screens in murine models have primarily used *P. chabaudi* and *P. yoelii* to understand blood-stage infections, and *P. berghei* to uncover determinants of resistance to cerebral malaria. A diversity of murine crosses, backcrosses, and congenic mice have been used for genetic linkage studies, for which C57BL/6, DBA/2J, and 129/SvJ possess the resistance phenotype, while A/J, NC/Jic, and BALB/c are commonly used as susceptible mice.

#### Plasmodium chabaudi

For classical genetic studies on the resistance to *P. chabaudi* the nomenclature created for associated genetic regions is ‘*Plasmodium chabaudi* resistant loci’ or *Char.* Currently, twelve *Char* regions have been described, most of them with potential candidate genes that explain the resistance phenotype observed in the screens, but only a few of them have been experimentally confirmed.

In the absence of genetic maps, early studies suggested that classical genetic approaches in *Plasmodium* using the AxB/BxA RI lines could be leveraged to understand host gene drivers of survival traits such as low parasitemia and splenomegaly (Stevenson and Skamene, 1985). With defined genetic markers, the first investigations of loci related to *P. chabaudi* resistance in mice appeared in 1997. Using genetic crosses between resistant C57BL/6 x susceptible C3H/He or SJL strains, two *P. chabaudi* resistance loci, *Char1* and *Char2*, were mapped to chromosomes 9 and 8, respectively. *Char1* contained candidate genes such as *Hp* (haptoglobin), *Trf* (transferrin), and *RBPI* (retinol-binding proteins), while *Char2* colocalized with a heritable blood group polymorphism called *Ea1* (erythrocyte surface antigen) but no gene candidates were noted (Foote et al., 1997). One of the events that triggers immunopathology in malaria is red blood cell rupture resulting in increased serum concentrations of free hemoglobin. The degradation of hemoglobin causes the release of heme, which increases host inflammation due to Fenton reactions mediated by its iron group (Mendonça et al., 2012). In response, hosts use haptoglobin to scavenge free hemoglobin, decreasing the inflammatory effects of this damage-associated molecular pattern (DAMP). Moreover, it has been described that serum haptoglobin is toxic for *P. falciparum* in *in vitro* assays (Imrie et al., 2004). *Ea1* is a phenotype without a corresponding gene, and describes agglutination reactions between wild-derived and inbred mice, grouping them into RBC surface antigens of A, B and AB (Foster et al., 1968). In humans, blood group antigens are known mediators of *Plasmodium* pathogenesis. It is well established that people with blood type O are protected against severe malaria, compared with A, B, and AB blood types. RIFIN polypeptides expressed on the surface of red blood cells infected with *P. falciparum* preferentially bind A antigens causing rosette formation and vascular sequestration (Goel et al., 2015), processes that correlate with malaria disease severity in humans. These observations underpin the suggested candidates found within *Char1* and *Char2*.

In search of causal genes, the group of Simon J. Foote further dissected two regions inside *Char2* using a panel of nine *Char2* congenic strains derived from crosses between C3H/He and C57BL/6 inbred lines, but no new candidates were presented (Lin et al., 2006). Similar crosses again re-discovered *Char2* located on chromosome 8, but suggested were other candidates including the genes *GypA* (glycophorin A), *Erp1* (erythrocyte protein 1), *Il15* (interleukin 15), and *Scvr* class A scavenger receptor, now known as *Msr1* (macrophage scavenger receptor 1) (Fortin et al., 1997). Glycophorins are sialoglycoproteins abundantly expressed on animal red blood cells and are directly involved in erythrocyte invasion by *Plasmodium*, acting as ligands for the parasite’s erythrocyte binding antigen (EBA) (Jaskiewicz et al., 2019). CD8 T cells are an essential component of the protective response against malaria liver stages and their maturation to memory cells is mediated by a complex set of signals. The magnitude of the central memory CD8 T cell response is dependent on IL-15 and its use in vaccine formulations can enhance protective immunity against a virulent challenge (Zarling et al., 2013; Parra et al., 2015). Apart from its involvement in maintaining naïve, effector, and memory T cells, other reports have documented that IL-15 is widely expressed by a diverse group of cells and can act as a modulator of defense against intracellular pathogens due to its proinflammatory nature (Lin et al., 2006; Patidar et al., 2016). This same group would later identify an H-2-linked QTL *Char3*, that correlated with parasitemia, further implicating the role of T cells in immunopathology and protection against *P. chabaudi* (Burt et al., 1999).

In 2001, *Char4* was identified in the Phillipe Gros laboratory by a series of F2 backcrosses between congenic mice bearing resistance loci from C57BL/6 backgrounds and susceptible A/J mice (Fortin et al., 2001), and was later refined using crosses between resistant C57BL/6J and susceptible A/J, C3H, and SJL strains, which identified a loss of function mutation in the gene *Pklr* (pyruvate kinase L/R) on chromosome 3 (Min-Oo et al., 2003). Malaria has been a strong selective force for human genome evolution (Kwiatkowski, 2005). Pyruvate kinase and G6PDH (glucose-6-phosphate dehydrogenase) deficiencies are the most common cause of non-spherocytic anemia and are also linked to protection against *P. falciparum* infections (Ayi et al., 2008; van Bruggen et al., 2015). PKLR is critical for glycolysis, converting phosphoenolpyruvate to pyruvate resulting in ATP production (Ayi et al., 2008). The production of energy mediated by PKLR is important for cell types that lack mitochondria, such as immature red blood cells. Reduced ATP availability in *Pklr* deficient erythrocytes likely explain the protection against *Plasmodium* sp. infections conferred by the *Char4* locus (Min-Oo et al., 2003). Continued work from the Gros laboratory described the existence of *Char10*, a region that controlled peak parasitemia and suppressed the effects of PKLR loss of function in a congenic line (AcB62). AcB62 carried the protective *Char4* variant, *Pklr^I90N^*, but was still susceptible to *P. chabaudi* challenge. *Char10* gene candidates included *Adam10* (ADAM metallopeptidase domain 10), which is essential for thymocyte development, *Csk* (C-Terminal Src Kinase), a negative regulator of Th1 responses, *Pias1* (protein inhibitor of activated STAT1), an inhibitor of interferon signaling, and *Pml* (promyelocytic leukemia protein), previously described as acting on hematopoietic differentiation (Min-Oo et al., 2010). Despite their description, *Adam10*, *Csk*, and *Pml* were not further explored in malaria pathogenesis. In a follow up study, the genetic modifier on *Char10* was again mapped to chromosome 9 using an F2 cross between AcB62 (*Pklr^I90N^*) and another PK deficient strain CBA/Pk (*Pklr^G338D^*), and its function correlates with improved erythroid development, thereby preventing the associated hemolytic anemia in the presence of PK-deficiency (Laroque et al., 2017).

*Char9* was described in 2007 using an F2 cross between the highly resistant congenic mouse line AcB55 and susceptible A/J mice*. Char9*, located on chromosome 10, was associated with regulating merozoite replication (Min-Oo et al., 2007). *Char9* was predicted to contain 77 annotated transcripts that were prioritized based on mRNA expression profiling in different mouse haplotypes and nucleotide sequencing. After the evaluation, the Vanin genes (*Vnn1*/*Vnn3*) were identified as the gene candidates for *Char9*. The protein pantetheinase, encoded by *Vnn1*/*Vnn3*, promotes tolerance to tissue damage by modulating the ability to cope with oxidative stress. Low levels of pantetheinase activity have been observed in patients with cerebral malaria (Pitari et al., 2000; Naquet et al., 2014), and erythrocytes from mice with pantetheinase insufficiency suffered from high oxidative stress and exhibited increased risk of severe malaria (Rommelaere et al., 2015). Treatment of mice with cysteamine, a metabolite product of pantetheinase, increases mouse survival by reducing *P. chabaudi* merozoite replication (Min-Oo et al., 2010). *Vnn3* was not expressed in the spleens of susceptible mice, and in A/J mice, an additional *Vnn3* nonsense mutation correlated with abolished enzymatic activity observed in both spleen and liver. Collectively, the data strongly implicates *Vnn3* as the causal agent of *Char9*.

Additional loci revealed by classical genetics include *Char5* and *Char6* on chromosome 5, and *Char7* on chromosome 17, which were discovered using Advanced Intercross Lines (AILs) (Hernandez-Valladares et al., 2004). Several candidate genes located in the regions of the *Char5* and *Char*6 were postulated: the membrane protein *Act1* (actin-related gene 1), *Ache* (acetylcholinesterase), *Cora1* (correlation in cytokine production 1), *Epo* (erythropoietin), *Hspb1* (heat shock 27-kDa protein 1), and *Ncf1* (NADPH oxidase sub-unit). The same group, again using AILs lines, described a new locus *Char8* on chromosome 11 containing some important genes such as Th2 cytokines (*Il3*, *Il4*, *Il5*, *Il13*), WNT-signaling pathway regulators (*Tcf7*), growth factors (*Csf2*, *Gdf9*), and the heat shock protein, *Hsp4* (Hernandez-Valladares et al., 2004). In humans, IL-4 is a pleiotropic cytokine that regulates B cell growth and immunoglobulin secretion (Luzina et al., 2012). Genetic studies revealed that specific polymorphisms of the *Il4* gene are related to elevated levels of anti-*Plasmodium* IgG antibodies and lower parasitemia in individuals with decreased malaria affection (Luoni et al., 2001; Tangteerawatana et al., 2009). Whether IL-4 or other Th2 cytokine genes underpins *Char8* is unknown. An X-linked *Char11* was described in 2011 by Laroque et al. using 25 inbred mouse strains that correlated with peak parasitemia and survival (Laroque et al., 2012). Recently, a preprint was uploaded in BioRxiv which reported the evaluation of *P. chabaudi* susceptibility using the Diversity Outbred panel (Gupta et al., 2021). Despite the evaluation of a high-diverse panel, no significant QTLs were detected. The highest QTL peak observed from this screen mapped to chromosome 8 and the temporary name of *Char12* was assigned, however, its LOD score did not reach significance.

#### Plasmodium berghei

In mouse models of experimental cerebral malaria (ECM) caused by *Plasmodium berghei*, inflammatory responses regulated by transcription factors, cytokines and signaling pathways that mediate pro-inflammatory responses are required for pathology (Longley et al., 2011), which is largely driven by T cells (Nitcheu et al., 2003; Villegas-Mendez et al., 2012). Before 2000, classical genetic studies of cerebral malaria were complicated due to the severe susceptibility of most laboratory mouse strains to the *P. berghei* ANKA strain. To address this problem, in 2002, Bagot et al. used a cross between susceptible C57BL/6J and a wild-derived strain (WLA) to study protection against ECM (Bagot et al., 2002). Two *P. berghei* resistance loci (*Berr* = ‘*Berghei resistance’*), *Berr1* on chromosome 1 and *Berr2* on chromosome 11, were identified and contained genes such as transforming growth factor β2 (*Tgfb2*). However, due to the large regions of *Berr1* and *Berr2* loci, the authors decided not to point out more candidates. Using a cross between C57BL/6 and wild-derived strains, a new resistance locus *Berr3* was later described by the same group. *Berr3* mapped to chromosome 9 and conferred resistance to *P. berghei* ANKA infection (Campino et al., 2005). Although no gene candidates for *Berr3* were put forth, the authors reinforced the importance of using wild-derived strains to understand genes involved in experimental cerebral malaria resistance.

Genetic linkage analysis using laboratory mice as founders produced other QTLs. For example, genetic linkage analysis of F2 intercrossed progeny from C57BL/6 and DBA/2 mice produced a region in the middle portion of chromosome 18 that had a strong association with protection from ECM. The candidate genes in that region were: *Csf1r* (colony-stimulating factor 1 receptor), *Pdgfr* (platelet-derived growth factor receptor), *Pdgfrb* (platelet-derived growth factor receptor, beta polypeptide), *Cd14* (CD14 antigen), and *Ii* (Ia-associated invariant chain, also known as CD74) (Nagayasu et al., 2002). A recent report described that PDGFRβ is a host receptor for TRAP (thrombospondin-related anonymous protein), a *Plasmodium* protein responsible for sporozoite motility and involved in host infection (Steel et al., 2021). CD14 is a leucine-reach-repeat surface protein (LRR) related to the extracellular LRR-portion of TLR pattern recognition receptors (PRRs). When CD14 detects microbial ligands, it can activate a potent inflammatory cascade, including the release of TNF-α and IL-1β. However, it is also involved in the induction of ECM and the regulation of parasite density (Oakley et al., 2009). CD74 is involved in the formation and transport of MHC class II peptide complex and acts as a receptor for MIF (macrophage migration inhibitory factor) (Schröder, 2016). In a murine model of vaccination to prevent malaria, the use of a modified version of CD74 increased antibody responses against *P. falciparum* and the suppression of MIF-CD74 signaling also protected mice against severe malaria by *P. berghei* ANKA (Fougeroux et al., 2019; Baeza Garcia et al., 2021). Whether these genes are causative for the chromosome 18 QTL is unclear.

Other classical genetic experiments revealed an association of the H-2 region with susceptibility to ECM (Ohno and Nishimura, 2004) and a region just distal to the H-2 locus that conferred partial resistance to *P. berghei* liver stage infection (Gonçalves et al., 2008). *Berr5*, located on chromosome 19, influenced ECM susceptibility (Berghout et al., 2010). The authors suggested that *Berr5* may regulate ECM by modulating the Th1/Th2 balance after *P. berghei* infection, but no candidate genes were tested. Finally, an FVB/NJ (susceptible) x DBA/2J (resistant) F2 cross revealed *Berr9*, which overlapped with the previously described QTLs, *Char1* and *Pymr* (below), that correlated with resistance to *P. chabaudi* and *P. yoelii* infections, respectively (Bopp et al., 2013). Nevertheless, genes in the region were described by the authors as unlikely to explain phenotypic differences observed between mouse strains.

#### Plasmodium yoelii

*P. yoelii* has been proposed as a malaria model for liver stage infection because sporozoites induce less inflammation in murine livers than *P. berghei* and produces more exoerythrocytic forms (Baer et al., 2007). Looking to determine genetic regions associated with survival to malaria, in 2001 Masahiko Nishimura’s group used a backcross between NC/Jic and 129/SvJ to map the ‘*Plasmodium yoelii* malaria resistance’ locus, *Pymr*, that associated with survival to *P. yoelii* infections (Ohno et al., 2001). Importantly, *Pymr* overlaps in the same region of *Char1* suggesting the convergence of a common genetic variant important in the resistance to multiple *Plasmodium* species. Whether the common variant of *Pymr*, *Char1*, and *Berr9* is haptoglobin remains to be experimentally verified in rodent malaria. However, given the recent association of hypo-haptoglobin as a risk factor for severe childhood malaria would suggest this to be the most likely candidate (Abah et al., 2018).

Although the present review is focused on the host side of the host-parasite relationship, the study published by Wu et al. presented a trans-species expression quantitative trait loci (ts-eQTL) analysis (Wu et al., 2015). The ts-eQTL technique aimed to identify genomic loci in the parasite (*P. yoelii yoelii* 17XNL and *P. yoelii nigeriensis* N67) that controlled specific host transcriptional responses, resembling the approach taken to discover the *T. gondii* kinase ROP16 (Saeij et al., 2007). A significant technical contribution of this study is the implementation of the ts-eQTL and use of genome-wide patterns of LOD scores (GPLSs), in addition to LOD scores, to detect host genes in related pathways. This study permitted the discovery of many unknown type I IFN regulators that were experimentally verified. For example, host expression of a group of IFN related genes (e.g. *Mx2*, *Irf7*, *Tgfb3*, *Stat2*) were controlled by three separate genetic loci in *Plasmodium yoelii*. Though many of these co-expressed genes were known to be involved with type I IFN responses many were not, including *Helz2* and *Lrp12* among others. From the set of genes hypothesized to play a role in the type I IFN pathway, fourteen genes (4 mouse and 10 human) were experimentally tested and their role as IFN regulators was confirmed. In addition, this result implies that many uncharacterized genes that grouped with the type I IFN GPLSs, might also regulate this pathway. Hence, ts-eQTLs, which integrate parasite genetic variation data to host gene expression, can be used to uncover host regulatory modules not previously described.

#### Plasmodium falciparum

In 1996, one of the first studies using genetic linkage was published by Rihet et al. to explore genetic aspects of malaria control in human populations located in Burkina Faso, West Africa (Rihet et al., 1998). A total of 153 siblings (sibs) from 34 families were studied and sib-pair linkage analysis verified the association between blood parasitemia and chromosome 5q31-q33, a region encoding several genes with immune function. A locus within the 5q31-q33, named *Pfil1* (*P. falciparum* infection level 1), was associated with controlling blood stage *P. falciparum* infection levels. *Pfil1* encompasses central Th1/Th2 immune response genes such as *IL4*, involved in the production of anti-*P. falciparum* antibodies, *IL12B* with a direct effect in promoting IFNγ responses, and *IRF1*, a key transcription factor that mediates the effects of IFNγ and is essential for activating neutrophils and macrophages to control plasmodial infections (Bouharoun-Tayoun et al., 1995).

#### Eimeria

*Eimeria* is a genus of gastrointestinal parasites that infect mainly ruminants, rabbits, and poultry. Infections by *Eimeria* can cause tremendous economic impact by reducing the productive capacity and growth of animals (Gilbert et al., 2020). Due to the economic impact of *Eimeria* infections, forward genetic strategies have been used to determine the genetic basis of protection and susceptibility, aiming to create genetic variants and poultry lines resistant to infection.

The first use of forward genetics to understand *Eimeria* infections in chickens was published in 2003. For QTL mapping, two broiler lines (sire and dam) were used to produce F1 and F2 populations, and susceptibility to *E. maxima* was inferred by oocyst shedding. Suggestive QTLs were found on chromosomes 1, 6, and 8, but only one in a region close to the marker LEI0101 (chromosome 1) was reported as significant (Zhu et al., 2003). A similar approach was undertaken to refine the LEI0101 QTL with additional flanking microsatellite markers. This analysis returned a second marker, LEI007, that was linked to LEI0101, but its exact physical location could not be confirmed due to discordant association between linkage-map and physical distances (Kim et al., 2006). In 2009, a study was published using an F2 genetic cross between resistant Fayoumi and susceptible White Leghorn chickens to map resistance QTLs to *E. tenella.* Bodyweight, growth, plasma coloration, hematocrit, rectal temperature, and lesions were traits measured for analysis (Pinard-van der Laan et al., 2009). A total of 21 chromosome-wide significant QTL were identified, which located to six *Gallus gallus* chromosomes, but no candidate genes were explored in this study. A similar screen was performed using Fayoumi x Leghorn F2 progeny and a medium-density SNP panel that revealed 31 QTLs (Bacciu et al., 2014). The authors pointed out that variation in QTL detection depended on the chosen statistical model and marker densities of the genomic region studied. Some, but not all candidate genes implicated were involved in immune responses to infectious diseases (*IFNγ*, *CCL20*, *IL22*, *IL2*, *CD4*), hematocrit levels, and others involved in carotenoid biosynthesis.

#### Sarcocystis

*Sarcocystis* is a genus of cyst-forming parasites that can use a diversity of mammals for sexual reproduction depending on the species of parasite. Following consumption of shed oocysts, dissemination leads to chronic infection with predilection for muscle tissue in the intermediate host. Symptomatic *Sarcocystis* spp. infection in humans is rare, but common in wild and domesticated animals, with potential economic impact on the cattle and pig industries (Fayer et al., 2015). Myotis, encephalitis, and abortion are associated with sarcoystosis in reared animals.

The first classical genetic screen for the *Sarcocystis* genus was published in 2007, which mapped QTLs affecting pig susceptibility to *Sarcocystis mischeriana* infection (Reiner et al., 2007). Meishan and Pietran pigs were crossed to produce F1 and F2 used to map traits. A total of 14 genome-wide QTL were identified. QTLs related to bradyzoite numbers (chromosome 7) and serum levels of *S. mischeriana*-specific IgG2 (chromosomes 7, 17 and X) were identified. The same author later published a series of QTL mapping results using the same model to study hematocrit, hemoglobin, red blood cell counts, mean corpuscular hemoglobin content (Reiner et al., 2007), white blood cell counts (Reiner et al., 2008), blood gases and pH (Reiner et al., 2009), and behavioral traits in swine and the first swine behavioral QTL analysis associated with a pathogen (Reiner et al., 2009).

### Genome-Wide Association Studies (GWAS)

#### Plasmodium

The first GWAS of Apicomplexa was published in 2008 by Jallow and collaborators screening for genetic correlates of severe malaria in Gambian patients (Jallow et al., 2009). A total of 2560 children were enrolled in the study, 1,060 severe malaria cases and 1,500 controls from rural and urban areas in Gambia. The strongest genome-wide association with severe malaria mapped to a region close to the hemoglobin *HBB* gene on chromosome 11p15, where the *HbS* polymorphism is located. Evidence for association was also reported for the genes *SCO1*, which encodes a protein involved in cytochrome oxidase function, and *DDC*, encoding dopa decarboxylase, which is involved in dopamine and serotonin synthesis. However, the association signal was attenuated due to weak linkage disequilibrium, indicating that the array used to study the population (Affymetrix 500K) lacked sufficient power to detect resistance loci with weak effects. The study’s main conclusion was that GWAS studies in African populations need a different approach than those typically used for European or Asian populations. The authors also suggested the development of an optimal genome-wide SNP genotyping platform for use in Africa, aiming to improve signal detection during GWAS.

In 2012 Idaghdour et al. published a combined GWAS and eQTL analysis of host-specific whole blood transcriptome signatures from children with non-complicated *P. falciparum* malaria living in Benin, West Africa (Idaghdour et al., 2012). The study found a strong genetic signature of genes associated with T cell activation and innate immunity such as *C3AR1* (complement component 3a receptor 1), *FCGR3B* (Fc gamma receptor IIIb), *RETN* (Resistin), *LRRC25* (leucine rich repeat containing 25), and *TAPBP* (TAP binding protein). Again, *SCO1* was found to be associated with infection, validating the aforementioned study by Jallow et al. Mammalian *SCO1* and *SCO2* are copper-binding proteins required for the assembly of cytochrome c oxidase (COX) in the mitochondria and contributing to the respiratory chain complex (Leary, 2010). The mechanism of action of SCO1 in malaria pathogenesis is still unclear, but the differential expression of *SCO1* regulating detoxification pathways of reactive oxygen species may contribute to *Plasmodium* control (Leary et al., 2009). Finally, to validate the findings, Idaghdour et al. infected C57BL/6 mice with *P. chabaudi* and compared gene expression with that observed in the human cohort and discovered 11 genes with the same pattern of response, including three Fc receptors (*Fcer2*, *Fcgr3b* and *Fcrla*) implicated in antibody-dependent phagocytosis and immune regulation. Overall, this study demonstrated the power of GWAS to detect genes related to infection and showed an interesting integration of GWAS, transcriptomics, and eQTL analysis from compatible datasets to evaluate genetic drivers of disease.

In 2012, Timmann et al. used GWAS to investigate a population in Ghana for correlates of parasitemia, hemoglobin, blood glucose, and lactate, and syndromes such as coma and respiratory distress that often develop with severe and/or cerebral malaria (Timmann et al., 2012). The study confirmed previous reports indicating an association between blood group O and sickle-cell traits with protection against malaria. In addition, two loci were associated with severe malaria. The first region mapped to chromosome 1q32, in the region of *ATP2B4*, an ATP-driven calcium pump that ejects calcium from the cell cytoplasm and regulates mean corpuscular volume and hemoglobin concentration in erythrocytes. The other region mapped to chromosome 16q22.2 in the region of *MARVELD3*, which encodes a tight junction protein. In a follow-up study, it was confirmed that *Atp2b4* controlled mouse susceptibility to cerebral malaria (Villegas-Mendez et al., 2021), highlighting how GWAS can lead to testable hypotheses surrounding host-parasite interactions.

Glycophorins are carbohydrate-containing proteins capable of binding lectins and are present in high quantities on the surface of animal red blood cells (Anstee and Tanner, 1986; Chasis and Mohandas, 1992). Early studies demonstrating that erythrocytes deficient in glycophorins were capable of resisting *P. falciparum* invasion helped reveal their role in malaria pathogenesis (Pasvol et al., 1982). In a multicenter GWA study evaluating severe malaria in populations from Gambia, Kenya, and Malawi, the Malaria Genomic Epidemiology Network reported a novel malaria resistance locus close to a cluster of genes encoding glycophorins (Jaskiewicz et al., 2019), which are host receptors for merozoite EBA and EBL invasion proteins (Sim et al., 1994; Mayer et al., 2009). The investigation also confirmed other loci previously associated with erythrocyte function and important for protection against malaria such as *HBB*, *ABO*, and *ATP2B4*, and that many of these alleles may have been under balancing selection that predated the human non-human primate split.

The study of immunogenic antigens in human malaria has been prolific. One of the questions frequently asked is whether antigen-specific responses to *Plasmodium* are influenced by the genetic background of the host and if so, what genes are responsible. One of the strategies proposed to fill this gap was published by Milet et al. (2016), in which they implemented GWAS for antibody reactivity to three common *P. falciparum* antigens (MSP1, MSP2, GLURP) detected in sera collected from children living in two villages in the Niakhar district of Senegal. A total of 174,950 SNPs were tested for association with IgG1 responses to these antigens and associations were found for 25 SNPs. The genes *RASGRP3* (RAS guanyl releasing protein), *RIMS1* (Rab-interacting molecules), *MVB12B* (multivesicular body subunit 12B), and *GNPTAB (*N-acetylglucosamine-1-phosphate transferase subunits alpha and beta) were pointed out as potential candidates, which function in various immune system processes such as the regulation of B cell proliferation, endosomal transportation, and lysosomal function.

In 2017, eQTL mapping and GWAS were combined to study *P. falciparum* malaria susceptibility and erythropoiesis-associated variables (Lessard et al., 2017). eQTLs were sought in *ex-vivo*-differentiated human erythroblasts, which are nucleated precursors to mature erythrocytes, focusing on genes presenting allelic imbalance (n=479). After genetic mapping and several enrichment steps, including positional cooccurrence of eQTL, SNPs, and open chromatin configurations within erythrocytes (Xu et al., 2012), the authors detected an erythroid-specific eQTL for the gene *ATP2B4*. Specifically, they identified 3 SNPs in an enhancer element defined by GATA1 and TAL1 binding, transcription factors required for normal erythrocyte development, which strongly correlated with *ATP2B4* expression. When deleted using CRISPR-Cas9, *ATP2B4* gene expression was drastically reduced, verifying the necessity of this enhancer element in *ATP2B4* gene expression. Finally, analyzing *Atp2b4^–/–^* mice, Lessard and collaborators confirmed that ATP2B4 was responsible for changes in mean corpuscular hemoglobin concentration and intracellular calcium levels. These results underpinned earlier GWAS which found the same SNPs to be associated with severe malaria (Timmann et al., 2012), and are consistent with the general role that osmolality and ion-dependent hydration have on intraerythrocytic parasitic growth (Tiffert et al., 2005). This study illustrates how the use of integrated approaches can facilitate the confirmation of genes responsible for resistance to parasite infection.

Signatures of immune function were also revealed by a GWAS published by Ravenhall et al. in which 914 individuals (449 patients and 465 controls) from the Tanga region in Tanzania were monitored for the presentation of four distinct types of malaria: hyperlactatemia, severe malarial anemia, respiratory distress, and cerebral malaria (Ravenhall et al., 2018). Correcting for the presence of the sickle cell *HbS* variant, a total of 53 SNPs were associated with protection. Specifically, *IL12BR2*, *IL23R*, and *KLHE* (kelck-like protein) presented genome-wide association, and a suggestive association was detected for certain HLA haplotypes. In 2019, Milet et al. published a GWAS in the context of non-severe malaria (Milet et al., 2019). 775 children in Benin were monitored from birth until 18-24 months of age for mild and recurrent malaria. The analysis revealed two association signals for mild malaria attacks located within the genes *SYT16* (synaptotagmin 16) and *PTPRM* (protein tyrosine phosphatase receptor type M). In addition, two signals for recurrent malaria were associated with the genes *ACER3* (alkaline ceramidase) and *PTPRT*, another receptor-type protein tyrosine phosphatase known to regulate lymphocyte signaling (Peyser et al., 2016).

GWAS has identified numerous SNPs associated with severe *Plasmodium* infections, however, only a few variant genes and their polymorphisms, such as *ATP2B4* and *HBB*, have been experimentally confirmed to be involved with malaria pathogenesis. Future validation of gene variants in malaria disease will clarify allelic specific drivers of malaria resistance.

#### Toxoplasma gondii

Acute and chronic *T. gondii* infections have been correlated with schizophrenia (Chorlton, 2017). A GWAS by Wang et al. tested the hypothesis that exposure to specific pathogens, including *T. gondii*, can increase the risk of schizophrenia or bipolar disorders and that genetic predisposition may underpin these disease states (Avramopoulos et al., 2015). The traits analyzed were antigen-specific IgG levels to common pathogens, as well as C-reactive protein (CRP), a peripheral marker of inflammation. The study failed to detect an association between these psychoses and infection, but *SGK1* (glucocorticoid-regulated kinase 1) and *SLC2A12* (solute carrier family 2) were suggested as plausible candidates associated with anti-*T. gondii* IgG levels. *SGK1* encodes an mTORC2-dependent regulator of the differentiation and function of T cells, which may influence resistance to *T. gondii*. In 2019, a similar GWAS was performed to identify genetic variants associated with *T. gondii* infection and its relationship to schizophrenia risk. Two groups of individuals were studied: an Ashkenazi cohort and a second group of predominately African Americans (Wang et al., 2019). No significant genome-wide associations were detected for predisposition to *T. gondii* and psychiatric disorders, but a suggestive SNP was found for *T. gondii* seropositivity in the region of the chitinase gene *CHIA*, previously associated with *T. gondii* brain cyst removal by macrophages (Nance et al., 2012). A similar GWAS of the same Ashkenazi cohort by Lori et al. did not find evidence for a correlation between schizophrenia and positive serology for *T. gondii*, but did note the genetic architecture of SNPs predicting schizophrenia are fundamentally different between seropositive and sero-negative schizophrenics (Lori et al., 2021). Although serological studies in humans are the most practical way to answer epidemiological questions regarding *T. gondii* infections (Dard et al., 2016), at best its use in GWAS has yielded marginal genetic associations for schizophrenia or susceptibility to infection. The lack of association probably has multifactorial origins.

#### Eimeria

*Eimeria* can negatively impact chicken maintenance and production. In 2015 the first GWAS was published for coccidiosis caused by *E. maxima* in Cobb500 Broilers, the most robust broiler in the world (Hamzić et al., 2015). 22 SNPs were detected across five chromosomes that correlated with body weight, plasma coloration, and β2-globulin content in blood plasma following *E. maxima* infection. Significant SNP associations were found for *THBS1* (thrombospondin-1), involved in angiogenesis and is highly expressed in the cecum of *Eimeria*-infected chickens, *FHOD3* (Formin Homology 2 Domain Containing 3), which regulates actin dynamics in cardiomyocytes, and other sugar remodeling enzymes, *MAN2C1* and *MGAT4C.* Collectively, these and other gene candidates suggest tissue repair and remodeling is central for protection against *Eimeria* infection in chickens.

Selective breeding can benefit from knowing genetic loci associated with reduced mortality to infection. In 2016, Psifidi et al. reported the use of GWAS for pathogen resistance in a group of African indigenous chickens in the western part of Ethiopia (Psifidi et al., 2016). A total of 760 chickens form Jarso and Horro ecotypes were analyzed for antibody titers to infectious bursal disease, Marek’s disease, fowl typhoid, fowl cholera and scored for resistance to *Eimeria* and cestode parasitism. Antibody titers to *Emeria* were associated with regions on chromosome 18 (5.5-6 Mb) and the MHC locus, the latter of which also correlated with resistance to cestodes. A follow up study by Banos et al. performed GWAS and whole-genome sequencing (WGS) on the same ecotype of chickens. Only variants of the gene *TOM1L1*, a signaling adaptor involved with Golgi and endosomal vesicular traffic, were associated with *Eimeria* infection (Banos et al., 2020). The same authors reported results from a GWAS to assess covariance and heritability of markers associated with *Eimeria* infection in commercial four-way crossbred Cobb500 Broilers (Boulton et al., 2018). Weight gain during infection, cecal lesion score, and the levels of IL-10, an immunosuppressive cytokine important in a variety of mouse models for inflammatory bowel disease, were individually measured. Only suggestive SNPs were detected for body weight (genes *FAM96B* and *RRAD*), but none for cecal lesions and IL-10. The authors discussed that based on the variance and extensive heterozygosity of the broilers, the study was underpowered to detect genome-wide associations controlling the response to *Eimeria*, reaffirming the importance of trait and organism selection for genetic studies.

Aiming to disentangle the genetic architecture controlling resistance to *E. maxima*, Boulton et al. published a GWAS study in chickens derived from two Leghorn inbred lines characterized by different susceptibility to *Eimeria* infection (Boulton et al., 2018). The study aimed to map genes associated with resistance to primary infection and a heterologous secondary challenge in progeny from an F2 intercross (C.B12 x 15I) and backcross [(C.B12 x 15I) x C.B12]. The traits measured were parasite replication, intestinal lesion score, and IL-10. The study found genome-wide association on chromosomes 1, 2, 3, and 5 for primary infection resistance and a suggestive association on chromosome 1 after a heterologous infection. Analysis of SNPs defining primary infection resistance revealed an enrichment for immune response genes, such as *Il6*, and genes involved in NF-κB and TLR signaling and confirmed IL-10 as a suitable marker disease severity.

#### Cryptosporidium

*Cryptosporidium* is an apicomplexan parasite that reproduces within the intestinal epithelium of mammals and can cause enteritis in both immunocompetent and immunocompromised individuals. In 2013 the Global Enteric Multicenter Study (GEMS) identified *Cryptosporidium* as an important pathogen associated with diarrhea in sub-Saharan Africa and South Asia, regions where death due to diarrheal disease is highly reported (Kotloff et al., 2013; Striepen, 2013). In 2020 the first and only GWAS of cryptosporidiosis in children was performed by Wojcik et al. (2020). The samples were collected from the Cryptosporidiosis birth cohort from Mirpur (urban) and Mizarpur (rural) communities in Bangladesh. Six SNPs in an intron of *PRKCA* (protein kinase C alpha) were associated with increased risk for symptomatic *Cryptosporidium* infections during the first year of life. Based on public eQTL databases, the authors hypothesized the existence of an association between *PRKCA* expression and inflammation mediated by Th17 cells. The importance of Th17 immunity to *Cryptosporidium* is largely unknown, though the IL-17 cytokine is induced in the murine intestine following infection (Zhao et al., 2016).

### Genome-Wide Linkage Analysis (GWLA)

#### Plasmodium falciparum

In 2007, a GWLA was published for the first time to identify genetic variants promoting resistance to malaria in human populations (Timmann et al., 2007). Screening 2,551 families in hyperendemic rural Ghana revealed associations with hemoglobin variants *HbS* and *HbC*, alpha^+^ thalassemia and *G6PD* deficiency with malaria resistance. These well-established variants inhibit the parasites’ ability to metabolize hemoglobin and maintain productive red blood cell infections. However, the region showing the strongest genetic association for fever responses during malaria mapped to the locus named ‘*P. falciparum*-fever episode 1’ (*PFFE-1*). *PFFE-1* maps to chromosome 10p15.3–10p14 containing candidate genes such as *IL2RA* (alpha chain of the interleukin-2 receptor), *IL15RA* (alpha chain of the interleukin-15 receptor), *GATA3* (GATA-binding protein 3), and *PFKP* (phosphofructokinase), that latter is expressed in red blood cells. Another GWLA study presented results comparing two Senegalese villages with differences in ethnicity, malaria transmission intensity, and endemicity (Sakuntabhai et al., 2008). Analyzed traits were the number of clinical episodes of malaria, parasite density, the frequency of asymptomatic *P. falciparum* infections, and the maximum *P. falciparum* parasite density during asymptomatic infections. In particular, the number of *P. falciparum* clinical attacks mapped to a single region on chromosome 5q31 in the two Senegalese populations. The 5q31 locus contains a cluster of Th2 (Ohashi et al., 2003; Petritus and Burns, 2008; Meyer et al., 2011) and Th1 genes (Mangano et al., 2008), previously implicated in protection against *Plasmodium* infections. In 2010, a GWLA using a 250K SNP map was reported by Milet et al. to study *P. falciparum* infection intensity and mild malaria in 626 individuals, 2-18 years old from Senegal (Milet et al., 2010). The analyses showed suggestive linkage for mild malaria attacks (chromosome 6p25.1 and 12q22) and prevalence of *P. falciparum* infection (chromosome 20p11q11) but no gene candidates were provided. A GWLA study conducted by Brisebarre et al. integrated data from two Burkina Faso regions (one urban and other rural) and was the first to integrate GWLA to detect genetic association with *P. falciparum-*specific antibody responses (Brisebarre et al., 2015). Since high parasite-specific IgG3 and low IgG4 levels were previously associated with malaria resistance (Aucan et al., 2000; Balogun et al., 2014), the chromosomal regions linked to IgG3 and IgG4 levels were sought. The analysis detected a significant linkage of parasite-specific IgG3 levels to chromosome regions 8p22-p21 and 20q13, and IgG4 levels to chromosome 9q34; however, no evidence of linkage to genes encoding cytokines was found.

#### Toxoplasma gondii

In 2015, a GWLA study was used to test the hypothesis that human populations differ in susceptibility to infections, including *T. gondii*, due to genetic variation. 428,000 SNPs in low linkage disequilibrium were used to correlate levels of pathogen-specific IgG to 12 different pathogens in Mexican American families in Texas (Rubicz et al., 2015). Significant linkage was observed for Human Herpesvirus 6 and 8 and Hepatitis A Virus, but no genetic association was found for anti-*T. gondii* IgG. Although not a GWLA, a GWAS study reported in 2018 (Scepanovic et al., 2018) also did not detect genetic association with seropositivity for *T. gondii*. The study was conducted on individuals from the *Milieu Intérieur* cohort in France, designed to understand the human immunological variance (Thomas et al., 2015). Non-genetic factors such as age (Wilking et al., 2016), geographic location (Pappas et al., 2009), and diet (Jones et al., 2009), are known to have a strong influence on *T. gondii* infection in humans and are likely to supersede the effect of any gene drivers of human susceptibility to this parasite.

### ENU Mutagenesis Screens

#### Plasmodium chabaudi

To our knowledge, ENU mutagenesis screens have only been used in the context of mouse *Plasmodium* infections. Since healthy red blood cells are required for productive parasite infections, a common strategy has been to first screen mutagenized mice for variables related to erythrocyte morphology, hemoglobin content, and abundance. Many identified genes from these screens impact merozoite asexual cycle, revealing the importance of red blood cell physiology to regulate parasite survival and maturation in a cell autonomous manner. Of note, most identified erythrocytic mutations are propagated and studied in mice as heterozygotes due to the early lethality observed in homozygous fetuses and pups.

Taking this approach, Rank et al. employed a large-scale ENU mutagenesis screen in mice to identify dominant mutations that affected erythroid production and maturation. From this screen an *Ank-1^1674^* null mutation was mapped by crossing affected heterozygous animals to C57BL/6, and the F2 from these crosses were studied using a genome-wide scan of sequence-length polymorphisms (SSLP) markers (Rank et al., 2009). ANK-1 is an erythrocyte cell membrane protein that has an essential role in the erythrocyte cytoskeleton structure. Homozygous *Ank-1^1674^* mutants exhibited severe anemia while heterozygotes had mild differences in red blood cell size compared to wildtype animals. Although the parasites could invade and mature within the red blood cells (RBCs) from *Ank-1^1674/+^* heterozygous animals, these mice were highly resistant to *P. chabaudi* infection by a mechanism associated with increased osmotic fragility of *Ank-1^1674/+^* erythrocytes. A similar ENU mutagenesis screen performed by Greth et al. in the Burgio laboratory discovered a dominant mutation in the *Ank-1* gene (*Ank^MRI23420^*) that produced mice with hereditary spherocytosis, a disorder in which RBCs form spheres instead of its normal concave structure (Greth et al., 2012). Mice heterozygous for the mutation showed greatly improved survival to *P. chabaudi* and *Ank^MRI23420/+^* RBCs thwarted parasite survival following invasion as inferred by increased TUNNEL staining of dead intracellular parasites. Highlighting the complex yet central role that Ank-1 plays in *Plasmodium* infections, two separate mutagenesis screens performed in Burgio laboratory again discovered Ank-1 as important for *P. chabaudi* pathogenesis. Huang et al. reported an *Ank-1^MRI6869/+^* mutation induced in the B6.BKS(D)-Lepr^db^/J background that resulted in increased osmotic fragility, reduction in mean corpuscular volume, and reduced deformability of mutant RBCs, phenotypes that mimic hereditary spherocytosis. In contrast to the *Ank^MRI23420/+^* mutation, parasite growth within *Ank-1* ^MRI6869/+^ erythrocytes proceeded normally, however these RBCs were resistant to *P. chabaudi* merozoite invasion resulting in significant protection from infection. In addition, the authors discovered that uninfected erythrocytes from the mutated mice were more quickly cleared from circulation during infection, implicating a ‘bystander’ mechanism for plasmodial elimination whereby fewer cells exist for the parasite to infect, lowering the probability for severe disease (Huang et al., 2016). In 2017, the same group would report two more *Ank* mutations from a mutagenesis screen in the SJL/J background (Huang et al., 2017). The first one, *Ank-1^MRI95845^* caused an amino acid substitution capable of promoting rapid clearance of infected RBCs. The second mutation, *Ank-1^MRI96570/+^*, produced a truncated protein capable of inhibiting merozoite maturation and survival inside the infected RBC. Both mutations produced a hereditary spherocytosis-like syndrome and were more resistant to parasite invasion. These results highlight the important role the RBC cytoskeleton has on *Plasmodium* infections and underscore the concept of “allelic heterogeneity” in malaria, whereby polymorphisms within a single gene can lead to varied resistance mechanisms. Whether *Ank-1* allelic heterogeneity in human populations has hidden an association between *Ank-1* and resistance to malaria is unknown. However, the prevalence of spherocytosis in malaria-endemic regions is not well studied, and only isolated cases have been reported (Spector and Metz, 1963; Ustun et al., 2003; Sangerman et al., 2008; Hassan et al., 2009).

Other ENU mutagenesis screens in the Burgio laboratory uncovered additional mutations that impact *Plasmodium* infections. In 2015, a missense mutation in the gene encoding the transferrin receptor, *Tfrc^MRI24910/+^* was identified that reduced TFRC surface expression on reticulocytes and erythroblasts (Lelliott et al., 2015). The primary function of TFRC is to import iron complexed to transferrin, and is essential for normal erythropoiesis, RBC morphology, and iron homeostasis. Erythrocytes from *Tfrc^MRI24910/+^* mice were characterized by smaller size and reduced cell hemoglobin, and spleens from these mice had lower non-heme iron levels. Though iron deficiency in human populations correlates with better protection against severe malaria (Murray et al., 1975; Nyakeriga et al., 2004; Kabyemela et al., 2008; Jonker et al., 2012; Gwamaka et al., 2012) and *Plasmodium* scavenges iron for optimal growth (Raventos-Suarez et al., 1982), *Plasmodium* parasites survived better in *Tfrc^MRI24910/+^* erythrocytes and these mice were slightly more susceptible to infection revealing the complex role iron plays in malaria pathogenesis.

In 2016, a mutagenesis screen helped identify a gain-of-function mutation in the protein AMPD3 (adenosine 59-monophosphate deaminase), which converts adenosine monophosphate (AMP) to IMP, thereby depleting AMP which can be converted to ATP (Hortle et al., 2016). In normal RBCs, AMPD3 activity is suppressed, but in *Ampd3^T689A^* erythrocytes this repression is relieved presumably due to the high enzymatic activity rendered by this mutation. In turn, *Ampd3^T689A^* RBCs have low levels of cellular ATP and high amounts of IMP, which in turn shortens their lifespan in half. Although parasite invasion rates were unaffected and parasite growth rates were increased by this mutation, *Ampd3^T689A/+^* mice were resistant to *P. chabaudi* infection. The authors reasoned that erythrocyte alterations observed in *Ampd3^T689A/+^* mice may lead to better elimination of the parasites due to the high erythrocyte turnover, implicating AMPD3 as a therapeutic target during *Plasmodium* infection.

Another mutagenesis screen in SJL/J mice generated two separate mouse lines with single-point mutations in β spectrin (*Sptb*) (*Sptb^MRI26194^* and *Sptb^MRI53426^*) that impacted resistance to *P. chabaudi* (Lelliott et al., 2017). Mice containing *Sptb^MRI26194^* and *Sptb^MRI53426^* mutations were characterized by small erythrocytes (microcytosis). Like mutations observed in AMPD3 and certain *Ank-1* mutants, mice with *Sptb^MRI26194^* and *Sptb^MRI53426^* mutations were less susceptible to *P. chabaudi* due to the rapid clearance of atypical erythrocytes, an effect independent of parasite invasion, maturation, or survival within RBCs. To confirm the impact of cytoskeletal alterations on *Plasmodium* infection observed in mutant *Ank-1* and *Sptb* mice, amino acid modifications to the binding site between β spectrin and ankyrin-1 were produced using CRISPR/Cas9. The modifications could recapitulate effects observed in the *Sptb^MRI26194^* and *Sptb^MRI53426^* mice, such as erythrocyte deformability and protection against *P. chabaudi* infection due to enhanced clearance of infected RBCs.

Finally, in 2020, Schnider et al. reported a mouse line with modest but significant resistance to *P. chabaudi*, but not to *P. berghei* and *P. falciparum*. The mutant line presented a non-sense mutation in the gene encoding for porphobilinogen deaminase (PBGD) (*Pbgd^MRI58155^*) (Schnider et al., 2020). PBGD is an enzyme of the heme biosynthesis pathway, present in elevated levels in diverse groups of tumors and its deficiency is associated to acute intermittent porphyria (AIP) and other metabolic diseases (Gibson et al., 1998; Greenbaum et al., 2003; Greenbaum et al., 2003) The authors discovered that erythrocytes from mutant mice presented a regular pattern of *P. chabaudi* invasion, but the parasites displayed reduced intraerythrocytic survival. The pattern of infection in *P. chabaudi* was not replicated in infections with *P. berghei*, likely due to the preference of this species to invade reticulocytes for its replication (Schnider et al., 2020).

#### Plasmodium berghei

The Gros laboratory has performed several ENU mutagenesis studies to explore gene drivers of cerebral malaria. At least four independent mutations were discovered that impaired the T cell response, which is detrimental during *P. berghei* infection. In 2012, Bongfen and collaborators first reported a mutation in the *Jak3* gene that generated a protective effect against cerebral malaria (Bongfen et al., 2012). Jak3 is an important signaling kinase for cytokine receptors using the common gamma chain (γc), including those for IL-2, -4, -7, -9. -15, -21, which regulate many aspects of thymic and hematopoietic development and functional differentiation of immune cells. The resistance to cerebral malaria in *Jak3* mutant mice was largely explained by impaired CD8^+^ T cell development and responses, as transfer of CD8 T cells from wild type mice restored susceptibility to the infected *Jak3* mutant line. Torre et al. later reported a mutant line for the gene, *Themis*, obtained from crosses between mutagenized C57BL/6J and wild type C57BL/10J mice that conferred resistance to ECM (Torre et al., 2015). *Themis* plays a regulatory role in positive and negative selection during late thymocyte development by regulating T cell receptor signaling (Lesourne et al., 2009). The authors reported major reductions in thymic and splenic CD4 and CD8 T cells in the mutant *Themis^I23N^* line. Moreover, peripheral T cells in these mice exhibited reduced proinflammatory cytokine production following infection, which in turn lessened cerebral malaria caused by *P. berghei*. Infected *Themis^I23N^* mice were further characterized by possessing an intact blood-brain barrier and reduced leukocyte infiltration into the brain, highlighting the importance of T cell-mediated inflammatory responses in cerebral malaria pathogenesis. A third ENU-derived mutation, impacting the ZBTB7B (ThPOK) zinc finger transcription factor, was identified that caused protection against cerebral malaria caused by *P. berghei* ANKA (Kennedy et al., 2020). ThPOK is the master regulator of CD4^+^ lineage commitment in the thymus, without which developing T lymphocytes instead commit to the CD8^+^ T cell lineage (He et al., 2005). Correspondingly, homozygous *Zbtb7b^R367Q^* mutants exhibited significant reductions of thymic and peripheral CD4 T cells, and during infection fewer brain infiltrating proinflammatory leukocytes were observed, confirming the role of CD4 T cell help in promoting ECM pathogenesis (Villegas-Mendez et al., 2012; Ham et al., 2015).

Whereas the previous three mutations had profound impacts on T cell development, thereby explaining the resistance to ECM due to general T cell deficiency, in 2014 a genome-wide ENU mutagenesis screen identified a mutation in the gene *Ccdc88b* (coiled-coil domain containing protein 88b) that corelated with protection against cerebral malaria and had no bearing on thymic development (Kennedy et al., 2014). CCDC88B is a microtubule interacting protein that associates with DOCK8 and promotes the migration and cytotoxic responses of various leukocytes (Fodil et al., 2017). Kennedy and collaborators demonstrated that CCDC88B is highly expressed in T cells and myeloid cells, and that its deficiency impaired T cell activation, cytokine production and proliferation in response to *P. chabaudi* infection and TCR stimulation. Finally, transfer of wildtype T cells into the *Ccdc88b* mutant line rescued susceptibility to ECM, again underpinning the central role of T cells in immune-mediated pathology surrounding cerebral malaria.

The attachment of *Plasmodium* to the brain microvasculature generates microthrombosis and neuroinflammation, potent triggers of cerebral malaria (Hansen, 2012). Torre and collaborators described a mutation in the deubiquitinase enzyme USP15 (*Usp15* ^L749R^), located on chromosome 10, capable of conferring resistance to ECM (Torre et al., 2017). The enzyme USP15 belongs to the ubiquitin-specific peptidase family and is involved in a diverse set of cellular functions, including membrane trafficking, mitochondrial homeostasis (Cornelissen et al., 2014; Jongsma et al., 2016), oncogenesis, and responses to viral infections (Eichhorn et al., 2012; Pauli et al., 2014; Zhang et al., 2015; Zou et al., 2015). The authors described that USP15 acted concomitantly with the E3 ubiquitin ligase TRIM25 generating an anti-inflammatory effect capable of blocking type I interferon and protecting mutagenized mice against ECM. Other work by Torre and colleagues revealed two novel resistance loci for *P. berghei*, *Berr6* on chromosome 4 (79.6–97.3Mb) and *Berr7* on chromosome 1 (~1, 41.4 Mb) that were generated by an ENU mutagenesis screen for recessive alleles that drive resistance to infection (Torre et al., 2013). *Berr6* regulated the rapid development of cerebral malaria which was further modulated by *Berr7* epistatically. Although the localization of *Berr6* and *Berr7* was given, no specific gene candidates were discussed.

Potassium signaling is an important mediator of inflammatory responses to infection (Qiu et al., 2002). Simon Foote and Gaetan Burgio’s group published a study based on a previous mutagenesis screen describing a mouse line with alterations in the K-Cl co-transporter type-1 (KCC1) (*Kcc1^M935K^*) (Brown et al., 2015) and was found to be more resistant to *P. berghei* infection (Hortle et al., 2019). Disease correlated with a significant accumulation of CD4^+^ T cells and high TNFα levels in the brains of infected mice, immunopathology that is clearly exacerbated by Th1 immune responses in cerebral malaria (Grau et al., 1987; Ghazanfari et al., 2018).

### RNA Interference Screens

#### Plasmodium berghei

Work from the Mota laboratory was the first to use RNAi to target host genes in a FGS for Apicomplexa. Their first experiment aimed to understand how host signaling pathways impacted *P. berghei* hepatocyte infection by freshly isolated sporozoites from the *Anopheles* mosquito (Prudêncio et al., 2008). Using three different siRNAs per gene, a high-throughput assay was designed to individually block the expression of 727 genes encoding proteins with known kinase activity and kinase-interacting proteins in Huh7 cells (a human hepatoma cell line). From this screen, *MET*, *PKCζ*, *PRKWNK1*, *SGK2*, and *STK35* were confirmed to promote *P. berghei* infection rates. Specifically, MET encodes a hepatocyte growth factor (HGF) receptor that is required for malaria infection, presumably by inhibiting apoptosis allowing enhanced sporozoite survival in the infected cell (Carrolo et al., 2003; Leirião et al., 2005). *PRKWNK1* is a kinase that controls kidney salt homeostasis, osmoregulation, and regulates adhesion and migration in CD4^+^ T cells (Köchl et al., 2016). Although the role of *PRKWNK1* has not been deeply investigated in infectious disease models, a study of the blood transcriptome of children with cerebral malaria revealed an upregulation of *PRKWNK1* in cerebral malaria patients (Boldt et al., 2019). The SGK2 kinase regulates a wide variety of ion channels and mediates cell signaling processes (Zambrowicz et al., 2003). That down-modulation of *PRKWNK1* and *SGK2* led to reduced infection indicates the potential need for osmotic balance in hepatocytes targeted by sporozoites. *TSTK35* is expressed in muscle and epithelial tissues and regulates the actin-myosin cytoskeleton in non-muscle cells (Vallenius and Mäkelä, 2002), which can impact *Plasmodium* infection (Carrolo et al., 2003). The ζ isoenzyme of protein kinase C (PKCζ) is a member of the atypical PKC subfamily, with involvement in multiple cellular functions (Hirai and Chida, 2003). Considering the role of PKCζ in pathological liver processes (Durán et al., 2004; Moscat et al., 2006), the authors decided to prioritize *in vivo* siRNA to knockdown of PKCζ; and demonstrated its role in promoting hepatocyte invasion by *P. berghei* sporozoites. This was the first functional genomic-driven study to identify new host factors affecting *Plasmodium* sporozoite infection.

Using a similar approach, the same group published a study to screen 206 siRNA capable of targeting 53 lipoprotein pathway associated genes after a *P. berghei* sporozoite infection (Rodrigues et al., 2008). After the screening and selection process, the class B, type I scavenger receptor (SR-BI, CD36) had the strongest impact on *P. berghei* infection. Additional suppression, overexpression, and *in vivo* and *in vitro* assays analyzing *P. berghei* and *P. falciparum* confirmed that SR-BI is required for *Plasmodium* sporozoite invasion. In addition to promoting invasion, the SR-BI receptor was required for intracellular parasite development into exoerythrocytic forms and the receptor was visualized adjacent to the parasitophorous vacuole after infection. Given the role of this scavenger receptor in lipid transport, Rodrigues and colleagues presented evidence that intra- compared to extra-cellular stores of lipids and cholesterol were more important for supporting hepatocyte infection, suggesting that SR-B1 is a promising candidate for malaria prophylaxis by potentially blocking the feeding of lipids to the maturing sporozoite. These studies were instrumental for later identification of the micronemal protein P36 as a ligand binding partner for SR-B1, which was required for *P. berghei* and *P. yoelli* hepatocyte invasion (Manzoni et al., 2017).

An alternative use of RNAi libraries was published by Raphemot et al. aiming to identify host druggable factors capable of inhibiting *P. berghei* sporozoite infection in hepatoma cells (Raphemot et al., 2019). A library of pooled siRNAs capable of testing 6,951 druggable human genes, including G protein-coupled receptors, kinases, phosphatases, transcription factors, nucleic acid binding molecules, and others, was used to identify genes involved in *P. berghei* invasion in HepG2 cells. After transfection, HepG2 cells were infected with *P. berghei* expressing a luciferase reporter, and the replication of the parasites was evaluated 48h post-infection. After screening and filtering steps, the three top candidates associated with reducing parasite levels were the genes *COPB2, COPG1*, and *GGA1.* To confirm the effect of gene silencing, the authors performed rescue experiments expressing siRNA-resistant gene constructs and demonstrated that sporozoite growth was restored in the complemented cell lines. *COPB2* and *COPG1* were visualized surrounding the parasitophorous vacuole and belong to the coatomer protein complex, which is responsible for the transport of vesicles between the Golgi apparatus and the endoplasmic reticulum (Feng et al., 2021). Moreover, members of the GGA1 family are vesicle coating proteins involved in regulating protein traffic between the trans-Golgi network and the lysosome (Bonifacino, 2004). The common identification of *COPB2*, *COPG1*, and *GGA1* suggests that vesicle transportation is a strong potential druggable target to control *Plasmodium* parasites, which are likely hijacking host vesicles to support its liver stage development. As an example, drug-based strategies that target host endosome formation have been used to control *Trypanosoma cruzi* invasion into peritoneal macrophages (Barrias et al., 2010), showing the potential of targeting host cellular mechanisms involved with membrane compartments to block parasites.

#### Plasmodium falciparum

In 2015 Egan et al. used *ex-vivo* cultured erythrocytes to run a forward genetics screen based on RNAi to identify host determinants of *P. falciparum* infection (Egan et al., 2015). To achieve knockdown of gene expression in mature erythrocytes, the siRNA library was introduced in hematopoietic progenitor cells prior to erythropoiesis and erythrocyte maturation. Terminally differentiated erythroblasts, which maintained the effects of gene knockdown, were infected with *P. falciparum* and screened for genes that could interfere with invasion or development within the infected erythrocytes. The screen revealed the molecule CD55 as a top candidate, a glycosylphosphatidylinositol-linked regulatory protein that protects cells from lysis by complement and is a receptor for several viral and bacterial pathogens (Nowicki et al., 1988; Storry et al., 2010). Analysis of both laboratory-adapted and clinical isolates of *P. falciparum* confirmed the requirement of CD55 for *Plasmodium* erythrocyte invasion and noted CD55 human polymorphisms enriched in individuals of African descent. Egan’s group would later describe how CD55 mediates internalization of *P. falciparum* and that this effect occurs after discharge of the parasite’s rhoptry organelles (Shakya et al., 2021).

#### Toxoplasma gondii

Gaji and collaborators in the Caruthers laboratory performed an RNAi screen to discover druggable targets to control early *Toxoplasma* invasion in human cells (Gaji et al., 2013). The authors validated the top 100 hits obtained in the primary screen and discovered 24 gene targets potentially involved with *T. gondii* host cell invasion within the first 3 hours of the assay. Among the candidates detected were those with protease activity, ubiquitin ligases, kinases, phosphatases, and ion channel regulators. Among the 24 candidates, six genes (*PTK9L*, *PHPT1*, *MAPK7*, *MYLIP*, *PTPRR*, and *PPIL2*) were confirmed to have antagonist effect on the cytoskeleton, affecting actin dynamics and promoting the formation of F-actin filaments, a phenotype associated with decreased cell invasion by *Toxoplasma*. Many intracellular pathogens modulate host cell actin to facilitate invasion (Sibley, 2004), a pivotal process for *Toxoplasma* invasion (Gonzalez et al., 2009).

Laura Knoll’s group screened an RNAi library targeting 18,200 human genes to discover host factors required for regulating *T. gondii* growth (Moser et al., 2013) and identified 19 genes associated with reducing parasite *T. gondii* replication in HeLa cells. A further investigation of the cell cycle genes *Wee1*, helicases *Ddx19* and *Ddx48*, nuclear export factor 1 (*Ncf1*) and tubulin-4q (*Tubb4q*), a member of the tubulin family of structural proteins, revealed that gene silencing was able to promote HeLa cell death and it was the primary reason explaining the decreased parasite growth rates. It was noted that DDX48, NXF1, and DDX19 are important for mRNA export and targeting such genes might damage protein synthesis, decreasing cell survival observed in the gene silenced cells.

Oxygen availability and the cellular mechanisms used to sense it fundamentally regulate metabolism and determine cell survival under low oxygen gradients (Wheaton and Chandel, 2011; Ortmann and Nathan, 2021). Hypoxia-inducible transcription factor (HIF-1) is part of a well-established core of components that mediate cellular responses to hypoxia (Semenza, 2012). Given that most tissue culture-based experiments occur at atmospheric levels of oxygen (21% O_2_), little is known regarding host factors that restrict intracellular pathogens at much lower physiological oxygen levels (3% O_2_). *T. gondii* infection induces HIF-1 activation and parasite growth in *HIF1a^-/-^* cells is severely blunted (Spear et al., 2006), suggesting the presence of HIF-1 dependent factors that promote parasite growth at physiological levels of O_2_. An RNAi screen developed by Ira Blader’s group aimed to identify these HIF-1 dependent host genes (Menendez et al., 2015). In the screen, host genes were hypothesized to be HIF-1 targets important for parasite growth if they were known to be regulated by HIF-1 and whose knockdown limited parasite growth at 3% O_2_ compared to 21% O_2_. The only gene that met these criteria was the host enzyme hexokinase 2 (*HK2*). They demonstrated that following infection the abundance and activity of HK2 is regulated by HIF-1, and that HK2 dissociated from the mitochondrial membrane at low oxygen levels which was required for optimal parasite replication. Hence, the HK2/HIF-1 axis could be therapeutically exploited to inhibit *T. gondii* establishment at an intracellular level.

#### Gene Overexpression Screens

The signal transducer and activator of transcription 1 (STAT1) is part of the JAK/STAT signaling pathway and is involved in cell growth inhibition, cell differentiation, and is known for its involvement in several immune-mediated diseases (Afkarian et al., 2002; Ma et al., 2011; O’Shea et al., 2013). In the context of *T. gondii* murine infections, the STAT1 transcription factor mediates the effect of IFNγ, including induction of the vacuole destructive IRGs (Howard et al., 2011) and upregulation of MHC class II molecules (Lüder et al., 2001). All clonal strains of *T. gondii* are capable of directly inhibiting STAT1-dependent transcription in the host nucleus (Rosowski and Saeij, 2012) through specific interactions with their exported dense granules TgIST (Olias et al., 2016) and TgNSM (Rosenberg and Sibley, 2021). Whether the host counteracts STAT1 inactivation by these parasite effectors is unknown. In 2015, Beiting et al. used a high throughput cDNA library with over 18,000 (human and mouse) genes to investigate whether the individual ectopic expression of genes could be capable of disarming *Toxoplasma*-dependent STAT1 inactivation (Beiting et al., 2015). The library was screened in a STAT1-responsive luciferase reporter cell line that was infected by *T. gondii* and then stimulated with IFN-γ. A set of nine genes was identified as unique transcriptional modulators capable of enhancing STAT1 signaling with high specificity. Due to the expression in the brain, the orphan nuclear receptor *TLX* was selected for further investigation and was confirmed that deficiencies in *TLX* were related to impaired STAT1-dependent IL-12 production and high parasite burden during chronic infection. This high-throughput overexpression genetic screen outlined a feasible approach to detect novel STAT1 regulators with high efficiency.

A recent report by Rinkenberger et al. (2021) described the use of an overexpression library encoding 414 IFN-γ induced human interferon-stimulated genes (ISG). The library was screened in a high-throughput system to identify genes involved in growth restriction of a type III (non-virulent) *T. gondii* strain in human cells. From this screen a phospholipase, *RARRES3*, interfered with parasite growth by reducing the size of the parasitophorous vacuole, which in turn promoted early egress of the parasites. This effect was dependent on the enzymatic activity of *RARRES3* and occurred independently of all known cellular death pathways. *RARRES3* encodes a multi-functional enzyme with phospholipase activity and is involved in palmitoylation of Wnt/β-catenin signaling molecules, with described roles in immunoproteasome regulation and metastasis suppression (Morales et al., 2014; Hsu et al., 2015; Anderson et al., 2017). It is tempting to speculate that *RARRES3* may deplete host stores of lipid droplets, for which *T. gondii* readily scavenges (Nolan et al., 2017), thereby decreasing vacuolar size and forcing egress. At first sight, premature egress could speed *T. gondii* invasion to neighboring cells, promoting dissemination. However, premature egress may also expose the parasites to the extracellular environment where antibodies (Souza et al., 2021) and complement await (Sikorski et al., 2020). *RARRES3* may also force the parasite outside the cell before it has adequately recharged its rhoptry organelles and other machinery required for invasion (Sánchez-Arcila and Jensen, 2022).

### CRISPR/Cas9 Mutagenesis Screens

#### Toxoplasma gondii

To date, only a few studies have attempted CRISPR/Cas9 library screens to interrogate the host response to parasite infection, indicating we will likely see more studies using this formidable technique soon. The first study using a genome-wide CRISPR/Cas9 screen to study host genes involved in the response against Apicomplexa parasites was published in 2019 by Wu et al. (2019). The study’s objective was to identify host dependency factors (HDF) critical for *T. gondii* infection but without affecting cell viability. A CRISPR/Cas9 library targeting 19,050 human genes and 1,864 human pri-miRNAs was screened, and 1,193 potential HDFs were identified. After a filtering process, seven genes (*CBLB*, *USP17L24*, *USP19*, *HDAC7*, *ULK1*, *PIM1*, and *ENPP5*) were pointed out as top candidates and were selected for further validation by siRNA knockdown and each were required for promoting optimal *T. gondii* growth. Ubiquitination is a post-translational modification that controls multiple steps in autophagy, a major lysosome-mediated intracellular degradation pathway (Grumati and Dikic, 2018) and several of the selected genes share ubiquitin functions by Gene Ontology including CBLB (Casitas B-Lineage Lymphoma Proto-Oncogene B), USP17L24 (Ubiquitin Specific Peptidase 17 Like Family Member 24), and USP19 (Ubiquitin Specific Peptidase 19). In addition, ULK1 (Unc-51 Like Autophagy Activating Kinase 1) is a serine/threonine kinase involved in several cellular processes including autophagy, formation of autophagosomes, and neuronal differentiation (Lin and Hurley, 2016). Considering the close relationship between ubiquitination and autophagy, and that autophagy has been described to contribute to *T. gondii* infection control (Liu et al., 2016; Subauste, 2021), it is possible the genes found in this screen are potential new key players in autophagic processes to regulate *T. gondii* infections. Another manuscript recently submitted in BioRxiv also reported a CRISPR screen to study the role of type I IFN-induced genes in human macrophage responses to *T. gondii* (Gossner et al., 2022). The authors identified and confirmed that *MAX*, *SNX5*, *F2RL2*, and *SSB* are potent IFNα-induced inhibitors of *T. gondii* infections in human THP-1 cells, as well as *AMPD3* as a positive regulator of *T. gondii* growth in resting conditions. In particular, the authors suspect that SNX5, with a known role in promoting membrane curvature and binding phosphatidylinositol 3-phosphate (PtdIns(3)P) (Mim and Unger, 2012), may seed early autophagic processes known to be important in cell autonomous control of *T. gondii* in human cells.

#### Other Preprints

A manuscript from the Striepen laboratory, uploaded to bioRxiv by Gibson et al., reported using a genome-wide CRISPR/Cas9 screen to discover host genes required for restricting *Cryptosporidium parvum* infection in a human intestinal epithelial cell line HCT-8. The screen revealed that removal of multiple players in the type III interferon pathway are important determinants of host resistance to *C. parvum* infection *in vitro* and *in vivo* (Gibson et al., 2021). Mice deficient for type III (*Il28ra^-/-^*) but not type I IFN (*Ifnar* ^-/-^) cytokine receptors were required for early resistance following infection. Confirmatory experiments revealed that IFN-λ production was dependent on TLR3 recognition, and that mice treated with IFN-λ exhibited reduced parasite load and could protect immune deficient mice (e.g. *Rag2^-/-^, IL2ra-/-* or *Ifng^-/-^*) from severe outcomes. This paper is an excellent example of how to extrapolate results from a cell autonomous immunity screen to meaningful *in vivo* observations related to parasitic infection.

A preprint uploaded in bioRxiv used a genome-wide CRISPR/Cas9 library containing 123,642 sgRNA to target 19,031 protein-coding genes and 1,864 microRNAs. The screen reported the identification of a host regulator of microtubule organizing centers (MTOCs), CENPJ (centromere protein J), that was required for restricting liver stage development of *P. yoelli* (Vijayan et al., 2021). In the absence of CENPJ enhanced microtubule association with the sporozoite vacuole was observed. CENPJ is a centrosome protein that participates actively in centriole organization and integrity (Cho et al., 2006; Kohlmaier et al., 2009). Considering the role of CENPJ in microtubule organization after *P. yoelii* infection, the authors demonstrated that *Plasmodium* manipulates the organization of Golgi-associated non-centrosome MTOCs to initiate the organization of microtubule formation around the parasitophorous vacuole during *Plasmodium* liver stages. A potential explanation for this association is that *Plasmodium* changes vesicle traffic to access host nutrients, as has been described for *T. gondii* in its ability to hijack multiple host vesicle compartments to support tachyzoite replication (Coppens and Romano, 2020).

## Conclusions and Future Directions

Over the years, forward-genetics approaches have been successfully leveraged to uncover novel loci and genes that determine the genetic basis of host-parasite relationships. A review based on an experimental approach to study a diverse group of organisms has certainly returned a diverse biology to comment upon. However, some trends can be gleaned, and future directives can be inferred regarding the use of FGS going forward.

The first impression is that while classical genetic screens and GWAS have returned many QTLs and suggestive SNPs, most gene candidates have yet to be verified, and it is less common to find within a single report the initial description of the phenotype, QTL and casual gene drivers underpinning the locus, though there are some notable examples (Min-Oo et al., 2003; Min-Oo et al., 2007; Souza et al., 2021). In contrast, providing causal linkage between gene and phenotype is more readily identified with mutagenesis or library screens. Yet even with RNAi or CRISPR-Cas9 screens, due to the sheer number of hits that are produced, many gene candidates remain to be validated or further characterized. Hence, it may be helpful for the field to compile existent information from all FGS reports to aid interpretation. For example, in the recent pre-print, Vijayan and colleagues noted that while there was little overlap at an individual gene level between RNAi and CRISPR screens for gene candidates regulating sporozoite infection by *P. berghei*, there was significant overall enrichment for shared host pathways that the targeted genes belonged to (Vijayan et al., 2021). We have attempted to compile this data in hopes of facilitating a more rapid lookup of suggested candidates (**Supplemental Table 1**) and firmly proven genes determined from these FGS approaches (**Table 1**). In our own cursory assessment, we noted cell autonomous screens often return gene candidates associated with vesicular traffic, for example *TOM1L1*, *CD36*, *CD55*, *CNPEJ*, *COPB2*, and *COPG1*, and factors putatively involved with autophagic processes, such as *SNX5, USP17L24, ULK1*, and *USP17L24*. Moreover, the convergence of multiple FGS upon a single gene candidate is likewise powerful in revealing its function and its extrapolation to other disease models. For example, *Ccdc88b* identified as a T cell regulator through ENU mutagenesis for resistance to *P. berghei* (Kennedy et al., 2014) led to establishing its association within the 11q13 region of Crohn’s patients (Fodil et al., 2017). Although no established pipeline exists for facilitating comparison between different FGS, integration of this information within curated databases such as GeneNetwork (genenetwork.org), which has extensive information from murine classical genetics screens but lacks gene candidate information from mutagenesis or library-based screens or compiled within the HostDB platform of VEuPathDB (veupathdb.org), could be potentially useful.

**Table 1:** Host genes revealed by forward genetic screens analyzing Apicomplexa infections.

Year	Parasite	Host	FGS	Traits	Confirmed Genes	References
1989 1995	*T. gondii*	AxB/BxA RIL, H-2a congenic (mice)	Classical genetics	Resistance to chronic infection, brain cyst numbers	MHCI *L^d^* of A/J mice	(McLeod et al., 1989) (Brown et al., 1995)
2003	*T. gondii*	(B10.Q/J × BALB/c) × B10.Q/J F1 backcross (mice)	Classical genetics	Susceptibility to infection, loss of IL-12 signaling	*Tyk2* loss of function mutation in B10.Q/J	(Yap et al., 2001) (Shaw et al., 2003)
2006 2014	*T. gondii*	LEWxBN, LWxF344 (rats)	Classical genetics	Resistance to infection, macrophage death in response to *T. gondii*	*Nlrp1* allelic variation (*Toxo1 locus*)	(Cavaillès et al., 2006) (Cavailles et al., 2014) (Cirelli et al., 2014)
2021	*T. gondii*	AxB/BxA RIL (mice)	Classical genetics	Resistance to secondary infection	*Nfkbid* required for immunity and humoral responses to *T. gondii*	(Souza et al., 2021)
2001 2003 2010 2017	*P. chabaudi*	AcB55 × DBA/2 F2 AcB55 × A/J F2 AcB61 × A/J F2 (mice)	Classical genetics	Resistance to blood stage infection	*Pklr* loss of function mutation (*Char4*, chr3), lower parasitemia, enhanced reticulocytosis	(Fortin et al., 2001) (Min-Oo et al., 2003) (Min-Oo et al., 2010) (Laroque et al., 2017)
2007	*P. chabaudi*	AcB55 × A/J F2 (mice)	Classical genetics	Resistance to blood stage infection	*Vnn3* (*Char9*, chr10) loss of expression promotes lower merozoite replication	(Min-Oo et al., 2007)
2015	*T. gondii*	BMDMs from AxB/BxA RIL (mice)	eQTL	Macrophage response to infection and stimulation	*Ddx1* regulates nitric oxide production	(Hassan et al., 2015)
2012 2017 2021	*P. falciparum*	Humans	GWAS, eQTL	Protection against severe falciparum malaria	*ATP2B4* (chr 1q32), polymorphisms regulate RBC hemoglobin concentration, slight protection against ECM in deficient mice	(Timmann et al., 2012) (Lessard et al., 2017) (Villegas-Mendez et al., 2021) (Timmann et al., 2007)
2012	*P. berghei*	C57BL/6J × C57BL/10J (mice)	ENU	Protection against ECM	*Jak3* missense mutation, impaired development and hypo-responsiveness of CD8^+^ T cells	(Bongfen et al., 2012)
2014	*P. berghei*	C57BL/6J × C57BL/10J (mice)	ENU	Protection against ECM	*Ccdc88b* null mutation decreases T cell activation, cytokine production and proliferation	(Kennedy et al., 2014)
2015	*P. berghei*	C57BL/6J × C57BL/10J (mice)	ENU	Protection against ECM	*Themis^I23N^* null mutation, impaired CD4^+^ and CD8^+^ T cell development	(Torre et al., 2015)
2019	*P. berghei*	mixed BALB/c and C57BL/6 background (mice)	ENU	Protection against ECM	*Kcc1^M935K^* mutation impairs CD4^+^ T cell accumulation in the brain	(Hortle et al., 2019)
2020	*P. berghei*	C57BL/6 (mice)	ENU	Protection against ECM	*Zbtb7b^R367Q^* mutation impairs CD4^+^ T cell development, reduced brain infiltrating proinflammatory CD4+ T cells	(Kennedy et al., 2020)
2015	*P. chabaudi*	SJL/J (mice)	ENU	Low mean corpuscular volume of RBC	*Tfrc^MRI24910^* lowers TFRC surface expression on RBCs, increased parasite survival in RBC, increased host susceptibility	(Lelliott et al., 2015)
2016	*P. chabaudi*	SJL/J (mice)	ENU	Macrocytic anemia	*AMPD3* loss of function mutation reduces ATP levels, shortening RBC lifetime leading to a faster parasite elimination and improved host survival	(Hortle et al., 2016)
2009 2012 2016 2017	*P. chabaudi*	*Mpl* ^-/-^ BALB/c SJL/J B6.BKS(D)-Lepr^db^/J (mice)	ENU	RBC associated variables: maturation, size, fragility, numbers	*Ank-1*, multiple mutations promote survival by modulating RBC physiology, all mutations increased osmotic fragility and reduced size of RBC, in addition: *Ank-1^1674/+^* *Ank^MRI23420/+^* decreased parasite survival in RBC; *Ank-1* ^MRI6869/+^ reduced RBC deformability, less merozoite invasion, increased RBC clearance in spleen; *Ank-1^MRI95845/MRI95845^* Less merozoite invasion, increased RBC clearance in spleen; *Ank-1^MRI96570/+^* less merozoite invasion and survival in RBC	(Rank et al., 2009) (Greth et al., 2012) (Huang et al., 2016) (Huang et al., 2017)
2017	*P. chabaudi*	SJL/J (mice)	ENU	Low mean corpuscular volume and hemoglobin concentration of RBC	*Sptb^MRI26194^*, *Sptb^MRI53426^* null mutations, less parasite invasion, increased RBC clearance, enhanced host survival	(Lelliott et al., 2017)
2020	*P. chabaudi*	SJL/J (mice)	ENU	Low mean corpuscular volume of RBC	*Pbgd^MRI58155^* decreased parasite survival in RBC	(Schnider et al., 2020)
2013	*T. gondii*	HeLa cells (human)	RNAi	Parasite invasion	*PTK9L*, *PHPT1*, *MAPK7*, *MYLIP*, *PTPRR* and *PPIL2* knockdown enhances actin filamentation thereby preventing invasion	(Gaji et al., 2013)
2013	*T. gondii*	HeLa cells (human)	RNAi	Parasite growth	*DDX48*, *NXF1*, and *DDX19* knockdown promotes host cell death thereby inhibiting parasite growth	(Moser et al., 2013)
2015	*T. gondii*	HeLa cells (human)	RNAi	Parasite growth in low O_2_ conditions	*HIF-1* dependent *HK2* activation required for parasite growth at low O_2_	(Menendez et al., 2015)
2008	*P. berghei*	Huh7 hepatoma cell line (human)	RNAi	Sporozoite infection	*PKCζ *inhibition impairs host cell invasion	(Prudêncio et al., 2008)
2008	*P. berghei*	Huh7 hepatoma cell line (human)	RNAi	Sporozoite infection	*Scarb1* (SR-BI, CD36) is required for parasite invasion and intracellular parasite development	(Rodrigues et al., 2008)
2015	*P. falciparum*	Hematopoietic progenitor cells, *ex-vivo* cultured erythrocytes (human)	RNAi	Parasite invasion	*CD55* promotes parasite invasion of RBCs	(Egan et al., 2015)
2019	*P. berghei*	HepG2, Huh7 cell lines (human)	RNAi	Parasite growth	*COPB2, COPG1*, and *GGA1* are required for vesicular trafficking to vacuole and optimal parasite growth	(Raphemot et al., 2019)
2019	*T. gondii*	U20S cells (human)	Over-expression	Overriding *Toxoplasma*-dependent STAT1 inactivation	*TLX* enhances IFNγ dependent gene expression, deficient mice are susceptible to chronic infection	(Beiting et al., 2015)
2021	*T. gondii*	A549 lung carcinoma cell line (human)	Over-expression	Parasite growth	*RARRES3* reduces vacuole size and promotes premature egress	(Rinkenberger et al., 2021)
Pre-print	*P. yoelii*	HepG2-CD81 hepatocellular carcinoma cell line (human)	CRISPR/Cas9	Regulators of microtube remodeling around the sporozoite vacuole	*CENPJ* interference facilitates sporozoite survival by increased localization of microtubules around vacuole	(Vijayan et al., 2021)
Pre-print	*C. parvum*	*HCT-8* adenocarcinoma colon cell line (human)	CRISPR/Cas9	Host cell survival to parasite infection	Type III interferon pathway and IFN-λ required for mouse resistance to infection	(Gibson et al., 2021)

The second observation is that despite significant methodological advances made in forward genetics, some FGS approaches are underutilized which have incredible potential to add novel insights to Apicomplexa biology. Due to their importance in human disease, host FGS for Apicomplexa are mainly restricted to *Plasmodium* and *Toxoplasma gondii.* And yet low representation or lack FGS studies exist for many species of Apicomplexa with human or zoonotic impact (*Eimeria*, *Sarcocystis*, *Babesia*, *Cytoisospora, Theileria*), and those that impact invertebrates, such as the gregarines. Here CRISPR-Cas9 holds great promise. Cell autonomous immunity screens can be performed on relevant cell lines that support parasite growth to uncover resistance genes specific to each species of Apicomplexa. Library screens may also be applied to solve longstanding issues surrounding the tissue culture of certain Apicomplexa (Hijjawi, 2010; Rueckert et al., 2019). For example, the genus *Gregarina* lacks an *in vitro* tissue culture system. Perhaps a library screen of insect cells for genes that regulate growth may open new models for analysis of these prevalent parasites of insects. Another important example is the lack of a practical continuous *in vitro* culture system for *P. vivax*, the most widespread human malaria parasite (Thomson-Luque et al., 2017). Because *P. vivax* merozoites prefer reticulocytes to grow and differentiate, no long-term culture system has yet been developed to overcome this problem, which has hindered research. FGS library screens may be useful to identify gene drivers of long-term reticulocyte maintenance, necessary for *P. vivax in vitro* experimentation (Gunalan et al., 2020). Furthermore, though CRISPR libraries have been designed to inactivate gene function, they could similarly be designed to insert specific changes in DNA sequences that mimic SNPs found in the host population. In this way, aiming to study the relationship between gene variants and parasite growth, a CRISPR-Cas9 FGS could be developed, for example, to confirm human GWA studies and *Plasmodium* growth in RBCs.

Another underutilized FGS tool is the recently available CC and DO panels for classical genetic screens in mice. In addition to screening these panels for novel resistance factors, Apicomplexa parasites without an established animal model or lacking one that recapitulates certain sequelae observed in natural disease, the CC could be screened following the example reported by Rasmussen and collaborators, who identified individual CC lines that reproduced the hemorrhagic fever caused by Ebola observed in humans (Rasmussen et al., 2014). To our knowledge, only one report using DO mice to study Apicomplexa infections has been uploaded as a preprint (Gupta et al., 2021). We expect more observations derived from these panels in future years.

In conclusion, forward genetics screens have shed tremendous light on the host response to Apicomplexa infections and have created opportunities for a new generation of researchers. Although lessons learned from *Plasmodium* and *T. gondii* research can be transferred to other Apicomplexa, this is not always the case. The search for new animal models, the application of FGS to underexplored Apicomplexa, the use of new high-throughput FGS *in vitro* systems and the integration of several types of forward genetic screens represent some of the most exciting challenges in the Apicomplexa field. We have outlined some future directives.

### Future Directives and Questions

- Implementation of CRISPR-Cas9 library screens for cell autonomous immunity against other less studied Apicomplexans.
- Will analysis of diversity outbred panels reveal new host resistance factors and mouse lines able to recapitulate Apicomplexa disease and infection?
- Can ENU mutagenesis screens be leveraged to study other RBC infecting Apicomplexa parasites, such as *Babesia* and *P. vivax*?
- Need for compiling both experimental methods and candidate genes revealed by FGS.

## Author Contributions

JS-A and KJ compiled and wrote the manuscript. All authors contributed to the article and approved the submitted version.

## Funding

KJ is funded by NIH grants R01AI137126 and R21AI145403.

## Conflict of Interest

The authors declare that the review was conducted and written in the absence of any commercial or financial relationships that could be construed as a potential conflict of interest.

## Publisher’s Note

All claims expressed in this article are solely those of the authors and do not necessarily represent those of their affiliated organizations, or those of the publisher, the editors and the reviewers. Any product that may be evaluated in this article, or claim that may be made by its manufacturer, is not guaranteed or endorsed by the publisher.

## Supplementary Material

Supplementary Table 1 Confirmed and suggestive gene candidates revealed by host forward genetic screens analyzing Apicomplexa infections. The information presented is grouped by the forward genetic approach, Apicomplexa parasite, year of description, type of host, traits analyzed for genetic screening, potential and confirmed candidates, and reference. Gene candidates listed are those emphasized by the authors and does not represent an exhaustive list of all suggestive candidates or gene ‘hits’ turned up by the various forward genetic screens.

## Abbrevations

CC, Collaborative Cross; CM/ECM, Cerebral malaria/Experimental Cerebral malaria; CRISPR-Cas9, Clustered Regularly Interspaced Short Palindromic Repeat-Cas9; DO, Diversity Outbred; ENU, N-ethyl-N-nitrosourea; eQTL, expression Quantitative Trait Loci; FGS, Forward Genetics Screens; F2, Generation 2; GWAS, Genome-wide Association Studies; GWLA, Genome-wide Linkage Analysis; LOD, Log of Odds; QTL, Quantitative Trait Loci; RNAi, RNA interference; SNP, Single Nucleotide Polymorphism; ts-eQTL, trans-species (host-parasite) expression quantitative trait locus.
