# Supplementary material for: Forward Genetics in Apicomplexa Biology: The Host Side of the Story
Source: Front Cell Infect Microbiol. 2022 May 12;12:878475. doi: 10.3389/fcimb.2022.878475 (PMC9133346; doi:10.3389/fcimb.2022.878475)
Supplement: Supplementary Table 1 — Confirmed and suggestive gene candidates revealed by host forward genetic screens analyzing Apicomplexa infections. The information presented is grouped by the forward genetic approach, Apicomplexa parasite, year of description, type of host, traits analyzed for genetic screening, potential and confirmed candidates, and reference. Gene candidates listed are those emphasized by the authors and does not represent an exhaustive list of all suggestive candidates or gene ‘hits’ turned up by the various forward genetic screens. [file Table_1.docx]

**Supplemental Table 1**

| **Year** | ***Parasite*** | **Host** | **FGS** | **Traits** | **Candidates** | **References** |
| --- | --- | --- | --- | --- | --- | --- |
| 1978 1989 1995 | *T. gondii* | AxB/BxA RIL,  H-2a congenic  (mice) | Classical genetics | Resistance to chronic infection, brain cyst numbers | MHCI *L^d^* of A/J mice | (Williams, Grumet, and Remington 1978) (McLeod et al. 1989) (Brown et al. 1995) |
| 2003 | *T. gondii* | (B10.Q/J × BALB/c) × B10.Q/J F1 backcross  (mice) | Classical genetics | Susceptibility to infection, loss of IL-12 signaling | *Tyk2* loss of function mutationin B10.Q/J | (Yap et al. 2001)  (Shaw et al. 2003) |
| 2006 2014 | *T. gondii* | LEWxBN, LWxF344  (rats) | Classical genetics | Resistance to infection, macrophage death in response to *T. gondii* | *Nlrp1* allelic variation (*Toxo1 locus*) | (Cavaillès et al. 2006) (Cavailles et al. 2014; Cirelli et al. 2014) |
| 2015 | *T. gondii* | BMDM from AxB/BxA RIL panel (mice) | eQTL | Macrophage response to infection and stimulation | *Gbp* chr3 locus affects parasite survival; *Arg1* in response to IL-4; *Ddx1* regulates nitric oxide production | (Hassan et al. 2015) |
| 2021 | *T. gondii* | AxB/BxA RIL panel (mice) | Classical genetics | Resistance to infection | *Nfkbid* required for immunity and humoral responses to *T. gondii* | (Souza et al. 2021) |
| 1997 | *P. chabaudi* | C57BL/6 x susceptible C3H/He or SJL strains (mice) | Classical genetics | Resistance to infection | *Char1,* chr9(*Hp*, *Trf*, *RBPI*);  *Char2,* chr8(*Ea1*, *GypA*, *Erp1*, *Il15,* *Msr1)* | (Foote et al. 1997) (A. Fortin et al. 1997) |
| 1999 | *P. chabaudi* | C3H/He x C57BL/6 (mice) | Classical genetics | Resistance to infection | *Char3* (*H-2*) | (Burt et al. 1999) |
| 2001 2003 2010 2017 | *P. chabaudi* | AcB55 × DBA/2 F2  AcB55 × A/J F2  AcB61 × A/J F2 (mice) | Classical genetics | Resistance to blood stage infection | *Pklr* loss of function mutation(*Char4,* chr3), lower parasitemia, enhanced reticulocytosis; *Char10* (*Adam10, Csk, Pias1,* *Pml*) | (Anny Fortin et al. 2001) (Gundula Min-Oo et al. 2003)  (G. Min-Oo et al. 2010, 9) (Aurélie Laroque et al. 2017) |
| 2007 | *P. chabaudi* | AcB55 × A/J F2 (mice) | Classical genetics | Resistance to blood stage infection | *Vnn3* (*Char9,* chr10) loss of expression promotes lower merozoite replication | (Gundula Min-Oo et al. 2007) |
| 2004 | *P. chabaudi* | (A/J x C57BL/6J) F1, AIL  (mice) | Classical genetics | Resistance to infection | *Char5* and *Char6,* chr5 *(Act1*, *Ache*, *Cora1*, *Epo*, *Hspb1*, *Ncf1);*  *Char7,* chr17;  *Char8,* chr11 *(Il3*, *Il4*, *Il5*, *Il13, Tcf7, Csf2*, *Gdf9*, *Hsp4*) | (Hernandez-Valladares, Naessens, et al. 2004)  (Hernandez-Valladares, Rihet, et al. 2004) |
| 2012 | *P. chabaudi* | Inbred mouse strains and SM/J x C57BL/6J F2  (mice) | Classical genetics | Survival and peak parasitemia | *Char11,* chrX | (A. Laroque et al. 2012) |
| 2002 | *P. berghei* | C57BL/6J x wild-derived strain (WLA) (mice) | Classical genetics | Resistance | *Berr1* and *Berr2,* chr11 (*Tgfb2*) | (Bagot et al. 2002) |
| 2005 | *P. berghei* | C57BL/6J x WLA (mice) | Classical genetics | Resistance | *Berr3,* chr9 | (Campino et al. 2005) |
| 2002 | *P. berghei* | F2 C57BL/6 x DBA/2 (mice) | Classical genetics | Protection to ECM | chr18 (*Csf1r*, *Pdgfr*, *Pdgfrb, Cd14, CD74*) | (Nagayasu et al. 2002) |
| 2004 | *P. berghei* | CBA (mice) | Classical genetics | Susceptibility to ECM | *H-2* | (Ohno and Nishimura 2004) |
| 2008 | *P. berghei* | (C57BL/6 X BALB/c) F2 and B6.C-H2^d^/bByJ (mice) | Classical genetics | Partial resistance | Locus proximal to *H-2* | (Gonçalves et al. 2008) |
| 2010 | *P. berghei* | (C57BL/6 x BALB/c)F2 (mice) | Classical genetics | Resistance to ECM | *Berr5,* chr19 | (Berghout et al. 2010) |
| 2013 | *P. berghei* | FVB/NJ (susceptible) (mice) x DBA/2J (resistant) F2 (mice) | Classical genetics | Survival | *Berr9* (overlap with *Char1* and *Pymr*) | (Bopp et al. 2013, 9) |
| 2001 | *P. yoelli* | Backcross between NC/Jic and 129/SvJ (mice) | Classical genetics | Survival | *Pymr* | (Ohno et al. 2001) |
| 2015 | *P. yoelii* | C57BL/6 (mice) | ts-eQTL | Type I IFN response genes that were co-regulated by 3 independent parasite loci | type I IFN regulators: known (*Oas1g, Tgfb3, Tnfrsf12a, Stat2, Parp14, Oas1a, Adar1, Mx2, Irf7, S1pr5, Oas2, Dhx58, Ifit3, Usp18, Isg15, Ifi35*) and unknown (*Ak3*, *Fosl1*, *Inpp4a*, *Havcr2*, *Fcgr1*, *Bc016423*, *S1pr5*, *Parp14*, *Satb1*, *Selenbp2*, *Helb*, *Helz2*, *Lrp12*) | (J. Wu et al. 2015) |
| 1998 | *P. falciparum* | Human (sub-Saharan Africa) | Classical genetics | Blood parasitemia | *Pfil1,* 5q31-q33 (*IL4*) | (Rihet et al. 1998, 33) |
| 2003  2006 | *E. maxima* | Broiler chickens (sire and dam) | Classical genetics | Susceptibility (oocysts shedding) | chr1 *LEI0101* | (Zhu et al. 2003) (Kim et al. 2006) |
| 2009  2014 | *E. maxima* | F2 resistant Fayoumi x susceptible White Leghorn chickens | Classical genetics | Susceptibility (body weight, growth, plasma coloration, hematocrit, rectal temperature, and cecal lesions)  hematocrit counts, and others involved in carotenoid biosynthesis | 21 chr-wide QTLs (*IFNG*, *CCL20*, *IL22*, *IL2*, *CD4*) | (Pinard-van der Laan et al. 2009)  (Bacciu et al. 2014) |
| 2007  2008  2009 | *Sarcocystis* | Pigs (Pietran x Meishan) | Classical genetics | Susceptibility,  hematological variables, blood gases and pH, behavior | 14 genome-wide QTLs: bradyzoite numbers (chr7); anti-*S. mischeriana* (chr 7, 17, X) | (Gerald Reiner et al. 2007)  (G. Reiner et al. 2007)  (G. Reiner et al. 2008)  (G. Reiner, Fischer, et al. 2009)  (G. Reiner, Köhler, et al. 2009) |
| 2009 | *P. falciparum* | Human (Gambian patients) | GWAS | Severe malaria | Region close to *HBB,* *SCO1, DDC* | (Jallow et al. 2009) |
| 2012 | *P. falciparum* | Human (Benin) | GWAS and QTL | Non-complicated *P. falciparum* malaria, whole transcriptome analysis | *C3AR1, FCGR3B, RETN*, *LRRC25*, *TAPBP, SCO1* | (Idaghdour et al. 2012) |
| 2012  2017  2021 | *P. falciparum* | Humans (Ghana) | GWAS, eQTL | Protection againstsevere falciparum malaria | *ATP2B4* (chr 1q32), polymorphisms regulate RBC hemoglobin concentration, slight protection against ECM in deficient mice;  *MARVELD3* mapped near to chr 16q22.2 | (Timmann et al. 2012)  (Villegas-Mendez et al. 2021)  (Lessard et al. 2017) |
| 2016 | *P. falciparum* | Human(Niakhar district in Senegal) | GWAS | Anti- *P. falciparum* antibodies to MSP1, MSP2, and GLURP | *RASGRP3*, *RIMS1*, *MVB12B*, *GNPTAB* | (J. Milet et al. 2016) |
| 2018 | *P. falciparum* | Human (Tanga region in Tanzania) | GWAS | Hyperlactatemia, severe malarial anemia, respiratory distress, and cerebral malaria | *IL12BR2,* *IL23R,* *KLHE* | (Ravenhall et al. 2018) |
| 2018 | *P. falciparum* | Human (children in Benin) | GWAS | Mild malaria | Mild malaria (*SYT16, PTPRM*);  Recurrent malaria (*ACER3, PTPRT*) | (Jacqueline Milet et al. 2019) |
| 2015 | *T. gondii* | Human(Mexican American population),  Human (France) | GWAS | IgG reactivity to antigens from 12 common infections | No associations with *Toxoplasma* | (Rubicz et al. 2015)  (Thomas et al. 2015) |
| 2015 | *T. gondii* | Human | GWAS | *T. gondii* and risk for schizophrenia or bipolar disorders (antigen-specific IgG levels to common pathogens, including *T. gondii*, as well as C-reactive protein , a peripheral marker of inflammation) | No associations for schizophrenia;  *SGK1* and *SLC2A12* suggested as candidates for susceptibility to *T. gondii* | (Avramopoulos et al. 2015) |
| 2019 | *T. gondii* | Human (Ashkenazi cohort and a second group of predominately African Americans) | GWAS | Anti-*T. gondii* IgG | No associations for schizophrenia; *CHIA* for *Toxoplasma* infection | (Wang et al. 2019)  (Lori et al. 2021) |
| 2015 | *E. maxima* | Cobb500 Broilers | GWAS | Body weight, plasma coloration and β2-globulin content in blood plasma | *THBS1, FHOD3,* *MAN2C1, MGAT4C* | (Hamzić et al. 2015) |
| 2016 | *E. maxima* | African indigenous chickens in the western part of Ethiopia | GWAS | Antibody titers to pathogens including *Eimeria* | *TOM1L1*, region in chr18 (5.5-6 Mb), and the MHC locus | (Psifidi et al. 2016)  (Banos et al. 2020) |
| 2018 | *E. maxima* | Cobb500 Broilers | GWAS | Weight gain during infection, cecal lesion score, levels of IL-10 | *FAM96B, RRAD* | (Boulton, Nolan, Wu, Psifidi, et al. 2018) |
| 2018 | *E, maxima* | Chicken Leghorn inbred lines intercross (C.B12 x 15I) and a backcross ((C.B12 x 15I) x C.B12) | GWAS | Primary infection and a heterologous secondary challenge with *E. maxima* (parasite replication, intestinal lesion score, IL-10) | Suggestive QTLs on chrs 1, 2, 3, and 5 for primary infection | (Boulton, Nolan, Wu, Riggio, et al. 2018) |
| 2020 | *Cryptosporidium* | *Human* (Cryptosporidiosis birth cohort from Mirpur (urban) and Mizarpur (rural) communities in Bangladesh) | GWAS | Symptomatic cryptosporidiosis | *PRKCA* | (Wojcik et al. 2020) |
| 2007 | *P. falciparum* | Humans (rural Ghana) | GWLA | Susceptibility to falciparum malaria | *HbS*, *HbC*, alpha^+^ thalassemia, *G6PDH;*  Description of PFFE-1 locus chr 10p15.3–10p14 (*IL2RA*, *IL15RA*, *GATA3*, *PFKP*). | (Timmann et al. 2007) |
| 2008 | *P. falciparum* | Human (Senegal) | GWLA | Number of clinical episodes of *P. falciparum*, parasitemia, prevalence of asymptomatic *P. falciparum*, *P. falciparum* parasitemia during asymptomatic infection | No associations | (Sakuntabhai et al. 2008) |
| 2010 | *P. falciparum* | Human (Senegal) | GWLA | Infection intensity and mild malaria | Mild malaria attacks (chr 6p25.1 and 12q22); prevalence of *P. falciparum* infection (chr 20p11q11) | (Jacqueline Milet et al. 2010) |
| 2015 | *T. gondii* | Human (Mexican-American populations) | GWLA | Pathogen specific IgG including *T. gondii* | No significant associations with *T. gondii* | (Rubicz et al. 2015) |
| 2015 | *P. falciparum* | Human (Burkina Faso) | GWLA | IgG subclasses anti-*P. falciparum* | Linkage of IgG3 (8p22-p21 and 20q13);  Linkage IgG4 (9q34) | (Brisebarre et al. 2015) |
| 2012 | *P. berghei* | C57BL/6J × C57BL/10J  (mice) | ENU | Protection against ECM | *Jak3* missense mutation, impaired development and hypo-responsiveness of CD8^+^ T cells | (Bongfen et al. 2012) |
| 2013 | *P. berghei* | C57BL/6J and 129S1/SvImJ (mice) | ENU | Protection against ECM | *Berr6,* chr4 and *Berr7,*chr1 | (S. Torre et al. 2013) |
| 2014 | *P. berghei* | C57BL/6J × C57BL/10J  (mice) | ENU | Protection against ECM | *Ccdc88b* null mutation decreases T cell activation, cytokine production and proliferation | (Kennedy et al. 2014) |
| 2015 | *P. chabaudi* | SJL/J  (mice) | ENU | Low mean corpuscular volume of RBC | *Tfrc^MRI24910^* lowers TFRC surface expression on RBCs, increased parasite survival in RBC, increased host susceptibility | (Lelliott et al. 2015, 1) |
| 2015 | *P. berghei* | C57BL/6J × C57BL/10J  (mice) | ENU | Protection against ECM | *Themis^I23N^* null mutation, impaired CD4^+^ and CD8^+^ T cell development | (Sabrina Torre et al. 2015) |
| 2016 | *P. chabaudi* | SJL/J  (mice) | ENU | Macrocytic anemia | *AMPD3* loss of function mutation reduces ATP levels, shortening RBC lifetime leading to a faster parasite elimination and improved host survival | (Hortle et al. 2016) |
| 2009  2012  2016  2017 | *P. chabaudi* | *Mpl*^-/-^ BALB/c  SJL/J  B6.BKS(D)-Lepr^db^/J  (mice) | ENU | RBC associated variables:  maturation, size, fragility,  numbers | *Ank-1,* multiple mutations promotesurvival by modulating RBC physiology, all mutations increased osmotic fragility and reduced size of RBC, in addition:  *Ank-1^1674/+^*  *Ank^MRI23420/+^*  decreased parasite survival in RBC  *Ank-1*^MRI6869/+^  reduced RBC deformability, less merozoite invasion, increased RBC clearance in spleen  *Ank-1^MRI95845/MRI95845^*  Less merozoite invasion, increased RBC clearance in spleen  *Ank-1^MRI96570/+^*  less merozoite invasion and survival in RBC | (Rank et al. 2009)  (Greth et al. 2012)  (Huang et al. 2016)  (Huang et al. 2017) |
| 2017 | *P. chabaudi* | SJL/J  (mice) | ENU | Low mean corpuscular volume and hemoglobin concentration of RBC | *Sptb^MRI26194^,* *Sptb^MRI53426^* null mutations, less parasite invasion, increased RBC clearance, enhanced host survival | (Lelliott et al. 2017). |
| 2019 | *P. berghei* | Mixed BALB/c and C57BL/6 background  (mice) | ENU | Protection against ECM | *Kcc1^M935K^* mutation impairs CD4^+^ T cell accumulation in the brain | (Hortle et al. 2019) |
| 2020 | *P. berghei* | C57BL/6  (mice) | ENU | Protection against ECM | *Zbtb7b^R367Q^* mutation impairs CD4^+^ T cell development, reduced brain infiltrating proinflammatory CD4+ T cells | (Kennedy et al. 2020) |
| 2020 | *P. chabaudi* | SJL/J  (mice) | ENU | Low mean corpuscular volume of RBC | *Pbgd^MRI58155^* decreased parasite survival in RBC | (Schnider et al. 2020) |
| 2008 | *P. berghei* | Huh7 hepatoma cell line  (human) | RNAi | Sporozoite infection | *PKC *inhibition impairs host cell invasion | (Prudêncio et al. 2008) |
| 2008 | *P. berghei* | Huh7 hepatoma cell line  (human) | RNAi | Sporozoite infection | *SR-BI* (*Scarb1*, CD36) is required for parasite invasion and intracellular parasite development | (Rodrigues et al. 2008) |
| 2015 | *P. falciparum* | Hematopoietic progenitor cells, *ex-vivo* cultured erythrocytes  (human) | RNAi | Parasite invasion | *CD55* promotes parasite invasion of RBCs | (Egan et al. 2015) |
| 2019 | *P. berghei* | HepG2, Huh7 cell lines  (human) | RNAi | Parasite growth | *COPB2, COPG1,* and *GGA1* are required for vesicular trafficking to vacuole and optimal parasite growth | (Raphemot et al. 2019) |
| 2013 | *T. gondii* | HeLa cells  (human) | RNAi | Parasite invasion | *PTK9L*, *PHPT1*, *MAPK7*, *MYLIP*, *PTPRR* and *PPIL2* knockdown enhances actin filamentation thereby preventing invasion | (Gaji, Huynh, and Carruthers 2013) |
| 2013 | *T. gondii* | HeLa cells  (human) | RNAi | Parasite growth | *DDX48*, *NXF1*, and *DDX19* knockdown promotes host cell death thereby inhibiting parasite growth | (Moser, Pollard, and Knoll 2013) |
| 2015 | *T. gondii* | HeLa cells  (human) | RNAi | Parasite growth in low O_2_ conditions | *HIF-1* dependent *HK2* activation required for parasite growth at low O_2_ | (Menendez et al. 2015) |
| 2019 | *T. gondii* | U20S cells  (human) | Over-expression | Overriding *Toxoplasma*-dependent STAT1 inactivation | *TLX* enhances IFNγ dependent gene expression, deficient mice are susceptible to chronic infection | (Beiting et al. 2015) |
| 2021 | *T. gondii* | A549 lung carcinoma cell line  (human) | Over-expression | Parasite growth | *RARRES3* reduces vacuole size and promotes premature egress | (Rinkenberger et al. 2021) |
| 2019 | *T. gondii* | Human Foreskin Fibroblasts (HFF) | CRISPR/Cas9 | Parasite inhibition | *CBLB*, *USP17L24*, *USP19*, *HDAC7*, *ULK1*, *PIM1*, and *ENPP5* | (S.-Z. Wu et al. 2019). |
| Pre-print | *P. yoelii* | HepG2-CD81 hepatocellular carcinoma cell line  (human) | CRISPR/Cas9 | Regulators of microtube remodeling around the sporozoite vacuole | *CENPJ* interference facilitates sporozoite survival by increased localization of microtubules around vacuole | (Vijayan et al. 2021) |
| Pre-print | *C. parvum* | *HCT-8* adenocarcinoma colon cell line  (human) | CRISPR/Cas9 | Host cell survival to parasite infection | Type III interferon pathway and IFNλ required for mouse resistance to infection | (Gibson et al. 2021) |
| Pre-print | *T. gondii* | THP1cells (human) | CRISPR/Cas9 | Type I IFN dependent parasite inhibition | *MAX*, *SNX5*, *F2RL2*, *SSB* | (Gossner, Raper, and Hassan 2022) |

**Supplemental Table 1. Confirmed and suggestive gene candidates revealed by host forward genetic screens analyzing Apicomplexa infections.** The information presented is grouped by the forward genetic approach, Apicomplexa parasite, year of description, type of host, traits analyzed for genetic screening, potential and confirmed candidates, and reference. Gene candidates listed are those emphasized by the authors and does not represent an exhaustive list of all suggestive candidates or gene ‘hits’ turned up by the various forward genetic screens.
